# Positive and biphasic extracellular waveforms correspond to return currents and axonal spikes

**DOI:** 10.1038/s42003-023-05328-6

**Published:** 2023-09-18

**Authors:** Shirly Someck, Amir Levi, Hadas E. Sloin, Lidor Spivak, Roni Gattegno, Eran Stark

**Affiliations:** 1 Sagol School of Neuroscience, Tel Aviv University, Tel Aviv, 6997801 Israel; 2 Department of Physiology and Pharmacology, Faculty of Medicine, Tel Aviv University, Tel Aviv, 6997801 Israel; 3 Sagol Department of Neurobiology, Haifa University, Haifa, 3103301 Israel

**Keywords:** Neural circuits, Action potential generation

## Abstract

Multiple biophysical mechanisms may generate non-negative extracellular waveforms during action potentials, but the origin and prevalence of positive spikes and biphasic spikes in the intact brain are unknown. Using extracellular recordings from densely-connected cortical networks in freely-moving mice, we find that a tenth of the waveforms are non-negative. Positive phases of non-negative spikes occur in synchrony or just before wider same-unit negative spikes. Narrow positive spikes occur in isolation in the white matter. Isolated biphasic spikes are narrower than negative spikes, occurring right after spikes of verified inhibitory units. In CA1, units with dominant non-negative spikes exhibit place fields, phase precession, and phase-locking to ripples. Thus, near-somatic narrow positive extracellular potentials correspond to return currents, and isolated non-negative spikes correspond to axonal potentials. Identifying non-negative extracellular waveforms that correspond to non-somatic compartments during spikes can enhance the understanding of physiological and pathological neural mechanisms in intact animals.

## Abstract (web summary)

In-depth analysis of extracellular waveforms from freely-moving mice identifies their source and properties, offering widespread implications for how to interpret and understand neural signals captured with extracellular probes.

## Introduction

Extracellular recordings have been employed extensively during the last century to monitor the electrical activity of multiple neurons^1,2^. During an action potential, the recorded waveforms depend on neuronal morphology, spike initiation site, and the relative position of the electrodes and the subcellular compartments^3–7^. Extracellular potentials recorded next to different compartments are expected to exhibit distinct polarities^8–10^. However, most studies focus on somatic signals, recorded as negative deflections in an un-inverted extracellular record^11–14^, and non-negative potentials are rarely reported in vivo.

For simplified neuronal morphologies, computational models show that the somatic inward currents during a spike are balanced by outward return currents evident as positive extracellular spikes (P-spikes), expected to be generated next to the proximal dendrites^5,9,15,16^. P-spikes were reported to correspond to backpropagation of somatic spikes into the dendrites^17,18^ and to outward currents in the dendrites^19,20^. Others reported that P-spikes are generated by axonal potentials^21,22^. Near the axon, a propagating sodium spike is expected to yield inward currents with a negative phase, preceded by a wavefront of local return currents yielding a positive phase^9,15^. Together, the two phases are expected to generate a biphasic spike (B-spike). B-spikes were reported to correspond to axonal spikes^7,21,23–30^ or dendritic spikes^18,31^. When sodium spikes initiate at the distal dendrites, P-spikes are expected next to the soma, and B-spikes at the proximal dendrites^32,33^. Experimental studies suggested that B-spikes correspond to backpropagation of somatic spikes^34–36^.

Taken together, the experimental findings and computational models support three hypotheses for the source of non-negative extracellular potentials during action potentials: axonal forward propagation, dendritic backpropagation, and return currents. Thus, the compartmental origin of non-negative extracellular waveforms is not established. The three hypotheses may be contrasted in freely-moving animals using high-density extracellular recordings and network activity analysis in regions where the axo-dendritic axis is parallel to the recording array, the neocortex and hippocampal region CA1.

Sodium spikes generated at the axon initial segment (AIS) actively propagate forward along the axon and backwards into the soma and dendrites^37,38^. Therefore, in extracellular recordings near the soma, potentials generated by axons and backpropagation into the dendrites are expected to exhibit sub-millisecond time lags with respect to somatic spikes due to conductance time. Axonal potentials are expected to be narrower than the somatic spikes due to higher density of voltage-gated potassium (Kv1) channels in the AIS^39^, whereas backpropagating spikes are expected to be wider due to lower dendritic density of KA channels^40^. On the other hand, return currents in the proximal dendrites are expected to balance AIS spikes. Charge movement time, AIS-to-soma propagation delay, and the somatic charging add up to several dozens of μs, therefore return currents are expected to be narrow and occur in synchrony with or slightly before the somatic spikes. The opposite axo-dendritic orientation of pyramidal cells in neocortex and CA1 makes the comparison between the two regions informative. Furthermore, the laminar organization of CA1, the distinct place coding in pyramidal cells in CA1 and in CA3^41^, and the local generation of high frequency ripples, allow differentiating between local and non-local generation of non-negative spikes.

Here, we show that in CA1 and neocortex, near-somatic narrow positive extracellular potentials correspond to return currents, and isolated non-negative spikes correspond to axonal potentials.

## Results

### Positive extracellular spikes correspond to return currents

**Fig. 1:**
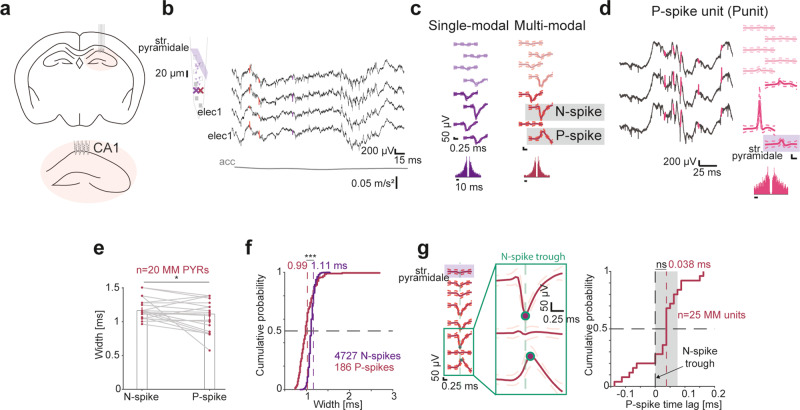
Positive extracellular spikes correspond to return currents. **a** Multi-shank recording array in hippocampal CA1 region. **b** Left, Schematic shank with 13 simultaneously-recorded units in CA1 of a freely-moving mouse. Str. pyramidale is in the top electrode. X marks correspond to the units depicted in (**c**). Right, Wideband (0.1–7,500 Hz) traces recorded by four adjacent electrodes. acc, head acceleration. **c** Single-modal (SM) and multi-modal (MM) units recorded simultaneously from CA1. Wideband spike waveforms and auto-correlation histograms (ACHs) of the SM pyramidal cell (PYR), and of the MM PYR with a negative spike (N-spike) and a positive spike (P-spike). **d** Positive unit (Punit) in extracellular recordings from CA1. A Punit is defined as a unit with a dominant positive peak in the main channel waveform. **e** P-spikes are narrower than same-unit N-spikes. Spike width, inverse of the dominant frequency of the waveform spectrum. MM PYRs with P-spikes and MM Punits are included. *p < 0.05, Wilcoxon’s test. **f** P-spikes of SM Punits are narrower than N-spikes of SM PYRs or MM PYRs with B-spikes. ***p < 0.001, *U*-test. **g** Same-unit N- and P-spikes unit occur in near synchrony. Left, The peak of the P-spike of a MM PYR occurs simultaneously with the N-spike trough. Right, The median time lag between N- and P-spikes in CA1 MM units is not consistently different from zero. ns, *p* > 0.05, Wilcoxon’s test comparing to a zero null. Gray patch, 95% confidence limits.

To determine whether spikes of multiple polarities are observable in the intact brain, we recorded 5971 units from hippocampal region CA1 of freely-moving mice (*n* = 9; Supplementary Fig. 1; Supplementary Table 1; Supplementary Table 2). Recordings were conducted using multi-shank arrays with vertical inter-electrode spacing of 15–20 μm (Fig. 1a).

Units with negative-spike polarity were recorded by one or more electrodes on the same shank, denoted as single-modal (SM) units (Fig. 1b, c). However, we also recorded units with spikes of distinct polarities on different electrodes, denoted as multi-modal (MM) units (Fig. 1b, c). Thus, in freely-moving mice, extracellular potentials of multiple polarities corresponding to a single spike are readily observable.

To determine whether extracellular P-spikes can be observed without N-spikes, we categorized the mean waveform on every electrode as an N-spike, a P-spike, a B-spike, or an undefined waveform according to the signal to noise ratio of the local extrema. Units for which the electrode with the largest trough-to-peak magnitude exhibited an N-spike were classified^42^ as pyramidal units (PYR) or PV-like interneurons (INT; Supplementary Fig. 1a). Units for which the positive peak was larger than the trough on the electrode with the largest trough-to-peak magnitude were denoted P-spike units, Punits. Units with a P-spike can be either SM Punits (only P-spikes; Fig. 1d), or MM Punits with a P-spike on the electrode with the largest trough-to-peak magnitude and N-spikes on one or more other electrodes. Furthermore, a unit with an N-spike can exhibit a P-spike on other electrodes (e.g., a MM PYR; Fig. 1c). Thus, P-spikes may be observed not only together with N-spikes, but also in isolation.

For a given unit, the spatial span is defined as the number of contiguous electrodes for which the waveforms are categorized, multiplied by the physical spacing between the electrodes. The spatial span of CA1 MM units with P-spikes was 160 [150 220] μm (median [interquartile interval, IQR]; *n* = 19), larger than the span of SM pyramidal cells (PYRs; 90 [60 100] μm; *n* = 4258; *p* < 0.001, *U*-test; Supplementary Fig. 1d). In contrast, the span of SM Punits was 40 [20 60] μm, smaller than the span of SM PYRs (*p* < 0.001) or interneurons (INTs; 95 [60 120] μm; *p* < 0.001, Kruskal–Wallis test corrected for multiple comparisons; Supplementary Fig. 1f). The magnitude of P-spikes was 81 [56 126] μV (*n* = 1434 spikes), smaller than the magnitude of N-spikes (114 [74 182] μV; *n* = 37255; *p* < 0.001; Supplementary Fig. 1g). Likewise, Punit waveform magnitude was 81 [56 126] μV, smaller than PYR magnitude (116 [76 187] μV) or INT (100 [66 154] μV; *p* < 0.001; Supplementary Fig. 1h). Punits were not recorded closer to the edge of the probes, compared with PYRs or INTs (Supplementary Fig. 1i,j). The signed distance between the electrodes on which same-unit N- and P-spikes were recorded was -30 [-125 125] μm (*n* = 25 MM units; Supplementary Fig. 1k**;** positive distances correspond to a P-spike above the N-spike). Thus, MM units which capture extracellular potentials corresponding to multiple neuronal compartments during a spike are readily observed in the intact brain.

Backpropagation into dendrites generates wider intracellular^40,43^ and extracellular^34,35^ waveforms compared with somatic spikes. To assess whether extracellular P-spikes correspond to backpropagation, we measured N- and P-spike widths using the inverse of the dominant frequency of the waveform spectrum. The width metric identifies the sinusoid frequency that has the best fit to the waveform shape, and is especially useful for signals with multiple extrema. P-spike widths were 1.11 [0.85 1.22] ms, whereas same-unit N-spikes were wider at 1.16 [1.07 1.28] ms (*n* = 20 MM units; *p* = 0.023, Wilcoxon’s test; Fig. 1e). Comparing the widths of N- and P-spike waveforms in CA1 regardless of the source unit, P-spikes widths were 0.99 [0.88 1.11] ms (*n* = 186 SM Punits), whereas N-spikes were wider (1.11 [1.07 1.16] ms; *n* = 4727 units; *p* < 0.001, *U*-test; Fig. 1f). Results were not sensitive to the choice of the waveform width metric (Supplementary Fig. 1m–p). N- and P-spikes were recorded in close proximity (up to 125 μm) in 75% of the cases (Supplementary Fig. 1k). Thus, P-spikes recorded in near proximity of N-spikes are unlikely to be produced by backpropagation of somatic spikes into the dendrites.

To contrast the possibility that P-spikes recorded in close proximity to the N-spike correspond to axonal potentials with the possibility of dendritic return currents, we measured time lags between the trough of N-spikes and the peak of same-unit P-spikes (Fig. 1g). The median [95% confidence limits] time lag between N- and P-spike in CA1 MM units was 38 [25 38] μs, not consistently different from zero (*n* = 25 MM units; *p* = 0.08, Wilcoxon’s test), and with an absolute value smaller than 50 μs (*p* = 0.041, Wilcoxon’s test; Fig. 1g). The timing analysis is correlative and does not demonstrate causality. Yet together, the waveform width and time lag analyses suggest that extracellular P-spikes that occur in close proximity to the soma correspond to return currents.

### Biphasic extracellular spikes correspond to axonal potentials

**Fig. 2:**
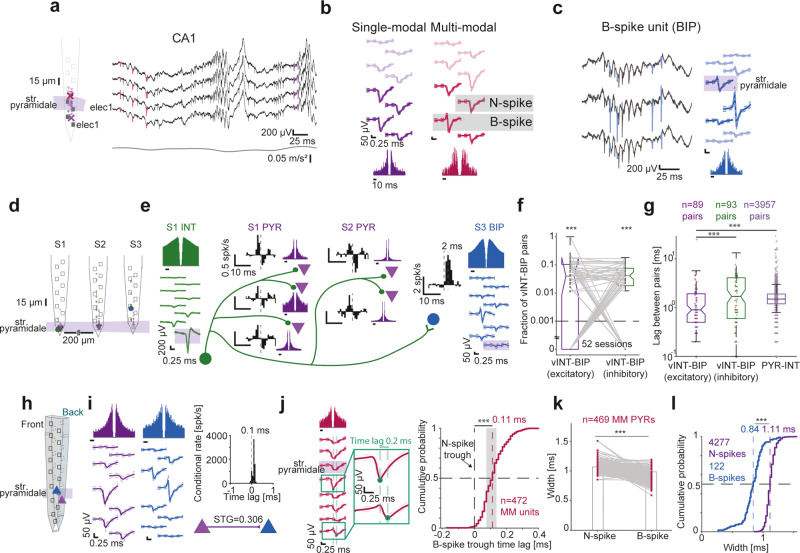
Biphasic extracellular spikes correspond to axonal potentials. **a** Left, Schematic shank with 32 simultaneously-recorded units in CA1 of a freely-moving mouse. Right, Wideband traces. All conventions here and in (**b**) are the same as in Fig. 1b, c. **b** Waveforms and ACHs of an SM PYR and an MM unit with biphasic spikes (B-spikes) recorded simultaneously from CA1. **c** B-spikes can appear without N-spikes. A biphasic unit (BIP) is defined as a unit with a dominant positive peak preceding a dominant negative peak in the main channel waveform. **d**, **e** A BIP exhibiting “monosynaptic excitation” by a verified inhibitory interneuron (vINT). **d** Probe schematic in CA1. **e** Derived network of a vINT, BIP, and five PYRs. Cross-correlation histograms (CCHs) show that the vINT is inhibitory for the five PYRs. However, the vINT-BIP CCH exhibits an “excitatory” monosynaptic peak, which can be accounted for by violation of Dale’s law or by a separate compartment of the vINT. Note similar vINT and BIP ACHs. **f** Fraction of vINT-BIP excitatory and vINT-BIP inhibitory connections in every session. Lined *p < 0.05, Wilcoxon’s test. Dashed line, chance level; ***p < 0.001, Binomial test comparing to chance level. Every box plot shows median and interquartile range (IQR), whiskers extend for 1.5 times the IQR in every direction, and a plus indicates an outlier. **g** Time lags for vINT-BIP excitatory connections, vINT-BIP inhibitory connections, and PYR-INT excitatory connections. Lined **/***p < 0.01/*p* < 0.001, *U*-test. **h**, **i** CA1 PYR-BIP pair recorded from opposite sides of a 30 μm dual-sided probe. The spike transmission gain (STG) corresponds to a 0.3 of a BIP spike occurring after a PYR spike. Transmission peaks at a sub-millisecond, one-sided time lag. **j** Time lag of B-spike trough relative to same-unit N-spike trough. ***p < 0.001, Wilcoxon’s test compared to zero. All other conventions are the same as in Fig. 1g. **k** B-spikes are narrower than same-PYR N-spikes. ***p < 0.001, Wilcoxon’s test. **l** B-spikes of SM BIPs are narrower than N-spikes of SM PYRs or MM PYRs with P-spikes. ***p < 0.001, *U*-test.

Action potential propagation in axonal and dendritic compartments was reported to generate triphasic spikes^18,24,31,36^, but using Butterworth filtering with a high-pass frequency of 300 Hz transforms B-spikes into triphasic spikes (Supplementary Fig. 2a). Because we used a very low cutoff frequency (0.1 Hz) and detrended the individual spikes (Supplementary Fig. 2b), previously-reported triphasic spikes likely correspond to B-spikes reported here. We recorded MM units with B-spikes in CA1 (Fig. 2a, b).

Every spike was quantified using a biphasic index (BPI) that measures the ratio between the positive and negative phases (Eq. 1). The BPI is -1 for a pure N-spike, 1 for a pure P-spike, and 0 for a symmetric B-spike (Supplementary Fig. 2c–f). Units for which the electrode with the largest trough-to-peak magnitude contained a B-spike are denoted as B-spike units, BIP (Fig. 2c). The signed distance between the CA1 electrodes on which same-unit N- and B-spikes were recorded was -40 [-60 -20] μm (*n* = 472 MM units; Supplementary Fig. 1l; positive distances correspond to a B-spike above the N-spike). Thus, B-spikes are observable in extracellular recordings of freely-moving mice.

Although some violations exist^44–47^, most neurons use the same neurotransmitter on all postsynaptic targets^48^. If distinct compartments of a purely-inhibitory neuron are recorded as two distinct units, the unit representing somatic potentials will appear to excite the unit representing non-somatic potentials. Cross-correlation histogram (CCH) analysis identified a CA1 network with seven units spanning three shanks that included an INT making inhibitory monosynaptic connections with five PYRs (Fig. 2d, e). We thus denote the INT as a verified inhibitory INT (vINT). The vINT made an apparently excitatory monosynaptic connection with a BIP (Fig. 2e). The vINT and the BIP could not have resulted from erroneously splitting a single unit during spike sorting, because the two units were recorded on distinct shanks, and because the CCH differs from the individual auto-correlation histograms (ACHs). The CCH peaked at a unidirectional 2 ms time lag, consistent with the 3 ms time lag expected from a conduction velocity of 0.13 ± 0.04 m/s in PV cell axons^49^ and an axonal potential triggered on a somatic spike ~400 μm away. The ACHs of the vINT and BIP had a rank correlation coefficient (cc) of 0.9 (*p* < 0.001, permutation test), whereas the median [range] cc’s between the ACHs of the vINT and the PYRs were 0.2 [-0.15,0.8]. The apparent contradiction of Dale’s law is resolved if the BIP corresponds to non-somatic spikes of the vINT.

vINTs and BIPs were recorded simultaneously during 52/126 (41%) of the CA1 recording sessions, yielding a total of 2208 vINT-BIP pairs of which 182 were connected (8.2%). The number of excitatory connections (89/2208 pairs, 4%) was above chance (0.1%; *p* < 0.001, Binomial test). The fraction of excitatory vINT-BIP pairs per session was 4% [1.7% 10%], not consistently different from inhibitory vINT-BIP pairs (4.1% [1.8% 7.6%]; *n* = 52 sessions; *p* = 0.43, Wilcoxon’s test; Fig. 2f). Any falsely-detected connection would diminish the observed differences. Moreover, time lags for “excitatory” vINT-BIP connections were 0.9 [0.5 1.9] ms (*n* = 89 pairs), shorter than for inhibitory vINT-BIP connections (1.7 [0.6 4.1] ms; *n* = 93; *p* < 0.001) and for excitatory PYR-INT pairs (1.5 [1.2 1.9] ms; *n* = 3957; *p* < 0.001, *U*-test; Fig. 2g). Thus, the network connectivity and timing analyses suggest that BIPs correspond to non-somatic potentials.

Independent support for the non-somatic origin of B-spikes comes from rare occasions in which two units corresponding to the same neuron were recorded simultaneously. In recordings from opposite sides of the same dual-sided shank (thickness, 30 μm; Fig. 2h), we found a PYR-BIP pair with extremely high spike transmission gain (STG; 0.306 postsynaptic spikes per presynaptic spike) and a CCH peak at a sub-millisecond, unidirectional time lag (Fig. 2i). The high gain and short time lag are inconsistent with synaptic transmission in freely-moving mice^50^. Thus, the network vINT-BIP and the dual-sided PYR-BIP analyses suggest that B-spikes correspond to recordings from a non-somatic compartment.

The dendritic backpropagation and axonal potentials hypotheses both predict that B-spikes will occur after initial spike generation. B-spike troughs followed the N-spike troughs (Fig. 2j) in 466/472 (99%) of the CA1 MM units, with median [95% confidence limits] lags of 110 [100 110] μs (*p* < 0.001, Wilcoxon’s test; Fig. 2j). Thus, the trough time lag analysis is inconsistent with dendritic return currents, and is in agreement with both backpropagation and axonal potentials.

In brain slices, dendritic backpropagating spikes are wider than somatic spikes^43^ whereas axonal spikes are narrower^39^. CA1 B-spikes had a width of 0.98 [0.91 1.02] ms, narrower than same-unit N-spikes (1.07 [1.02 1.11] ms; *n* = 469 MM units; *p* < 0.001, Wilcoxon’s test; Fig. 2k). Comparing the widths of B- and N-spikes regardless of the source unit, we found that B-spike widths were 0.84 [0.74 0.93] ms (*n* = 122 BIPs), whereas N-spikes were wider (1.11 [1.07 1.16] ms; *n* = 4277 units; *p* < 0.001, *U*-test; Fig. 2l). Thus, B-spikes recorded in near proximity of N-spikes are unlikely to represent backpropagation into the dendrites. Since both dendritic return currents and backpropagation are unlikely sources of B-spikes, axonal potentials are most likely.

### Neocortical non-negative extracellular spikes correspond to return currents and axonal potentials

**Fig. 3:**
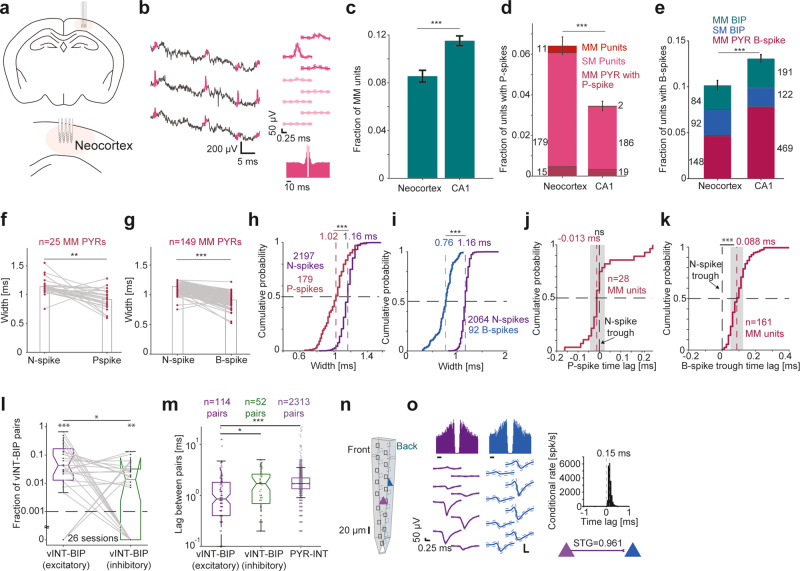
Neocortical non-negative extracellular spikes correspond to return currents and axonal potentials. **a** Multi-shank recording array in the parietal cortex. **b** Punit in extracellular recordings from the neocortex. **c** MM units are more prevalent in CA1 than in the neocortex. Dataset includes 3189 neocortical units from *n* = 17 mice and 5971 CA1 units from *n* = 9 mice. Here and in (**d**) and (**e**), ***p < 0.001, *G*-test; error bars, SEM. **d** Units with P-spikes are more prevalent in the neocortex than in CA1. **e** The fraction of units with B-spikes is higher in CA1 than in the neocortex. **f**, **g** P- and B-spikes are narrower than same-PYR N-spikes in neocortex. **/***p < 0.01/p < 0.001, Wilcoxon’s test. **h**, **i** P-spikes and B-spikes of neocortical SM Punits and BIPs, respectively, are narrower than N-spikes of SM PYRs or MM PYRs with P-/B-spikes. ***p < 0.001, *U*-test. **j** The median time lag between N- and P-spikes in neocortical MM units is not consistently different from zero. ns, *p* > 0.05, Wilcoxon’s test comparing to a zero null. Here and in (**k**) conventions are the same as in Fig. 1g. **k** Time lag of B-spike trough relative to same-unit N-spike trough. ***p < 0.001, Wilcoxon’s test compared to zero. **l** Fraction of vINT-BIP excitatory and vINT-BIP inhibitory connections in every session. Lined *p < 0.05, Wilcoxon’s test; **/***p < 0.01/*p* < 0.001, Binomial test comparing to chance level (dashed line). Box plot conventions are the same as in Fig. 2f. **m** Time lags for vINT-BIP excitatory connections, vINT-BIP inhibitory connections, and PYR-INT excitatory connections. Lined */***p < 0.05/ *p* < 0.001, *U*-test. **n**, **o** Neocortical PYR-BIP pair recorded from opposite sides of a 30 μm dual-sided probe. Spike transmission gain (STG) is close to unity. Transmission peaks at a sub-millisecond, one-sided time lag.

To generalize our findings in CA1, we repeated the experiments in an area with distinct network architecture, the parietal cortex (*n* = 3189 units from 17 mice; Fig. 3a; Supplementary Table 1). MM units, Punits, and BIPs were readily recorded in neocortex (Fig. 3b, Supplementary Fig. 3, Supplementary Fig. 4**;** Supplementary Table 3). Pooling the neocortex and CA1 datasets together, 966/9160 (10.5%) of the units were MM, with higher prevalence of MM units in CA1 (689/5971, 11.5%) compared with the neocortex (277/3189, 8.7%; *p* < 0.001, *G*-test; Fig. 3c). The fraction of units with P-spikes was higher in the neocortex (205/3189, 6.4%) compared with CA1 (207/5971, 3.4%; *p* < 0.001, *G*-test; Fig. 3d), whereas the fraction of B-spike units was higher in CA1 (782/5971, 13.1%) compared with the neocortex (324/3189, 10.2%; *p* < 0.001, *G*-test; Fig. 3e). Compared with P-spikes, B-spikes were more likely to be recorded with N-spikes (892 vs. 47 units, pooled over both regions; *p* < 0.001, *G*-test). Thus, extracellular potentials corresponding to multiple neuronal compartments during a spike are readily observed in both neocortex and CA1.

In accordance with the CA1 results, spatial spans of neocortical MM units with B-spikes were larger than the spans of SM pyramidal cells (*p* < 0.001, *U*-test; Supplementary Fig. 1c). In contrast, the span of neocortical SM BIPs and of SM Punits was smaller than of SM PYRs (*p* < 0.001) or INTs, similarly to CA1 (*p* < 0.001, Kruskal–Wallis test; Supplementary Fig. 1e). Furthermore, P-spike widths in neocortex were 0.91 [0.8 1.04] ms, whereas same-unit N-spikes were wider at 1.14 [1.09 1.16] ms (*n* = 25 neocortical MM units; *p* = 0.0011, Wilcoxon’s test; Fig. 3f). Neocortical B-spikes had a median [IQR] width of 0.91 [0.87 0.98] ms, narrower than same-unit N-spikes (1.14 [1.09 1.16] ms; *n* = 149 MM units; *p* < 0.001, Wilcoxon’s test; Fig. 3g). The widths of neocortical N-spike, P-spike, and B-spike waveforms regardless of the source unit were also similar to the CA1 results. Neocortical P-spikes widths were 1.02 [0.9 1.11] ms (*n* = 179 SM Punits), whereas N-spikes were wider (1.16 [1.11 1.19] ms; *n* = 2197 units; *p* < 0.001, *U*-test; Fig. 3h). Neocortical B-spikes widths were 0.76 [0.67 0.85] (*n* = 92 BIPs), whereas N-spikes were wider (1.16 [1.11 1.19] ms; *n* = 2064 units; *p* < 0.001, *U*-test; Fig. 3i). Thus, neocortical P- and B-spikes recorded in near proximity of N-spikes are unlikely to be produced by backpropagation of somatic spikes into the dendrites.

The timing analysis designed to test the correspondence of P- and B-spike to axonal potentials or return currents in CA1 yielded similar results in the neocortex dataset. The median [95% confidence limits] time lag between N- and P-spike in neocortical MM units was -13 [-19 0] μs, not consistently different from zero (*n* = 28 MM units; *p* = 0.91, Wilcoxon’s test), and with an absolute value smaller than 19 μs (*p* = 0.042, Wilcoxon’s test; Fig. 3j). B-spike troughs followed the N-spike troughs in 161/161 (100%) of the neocortical MM units, with median [95% confidence limits] lags of 88 [75 100] μs (*p* < 0.001, Wilcoxon’s test; Fig. 3k). Thus, the neocortex results support the interpretation that P-spikes occurring in close proximity to the soma correspond to return currents. Moreover, the results suggest that neocortical B-spikes are inconsistent with dendritic return currents, but are in agreement with axonal potentials.

vINTs and BIPs were recorded simultaneously during 26/80 (32%) of the neocortical recording sessions, yielding a total of 2495 vINT-BIP pairs of which 166 were connected (6.7%). The number of excitatory connections (114/2495 pairs, 4.6%) was above chance (0.1%; *p* < 0.001, Binomial test) and higher than the number of inhibitory connections (52/2495, 2.1%; *p* < 0.001, *G*-test). The fraction of excitatory vINT-BIP pairs per session was 4.3% [1.1% 17%], higher than inhibitory vINT-BIP pairs (1.4% [0% 3.2%]; *n* = 26 sessions; *p* = 0.016, Wilcoxon’s test; Fig. 3l). Moreover, time lags for “excitatory” vINT-BIP connections were 0.8 [0.4 1.8] ms (*n* = 114 pairs), shorter than for inhibitory vINT-BIP connections (1.7 [0.6 2.6] ms; *n* = 52; *p* = 0.019) and for excitatory PYR-INT pairs (1.7 [1.3 2.2] ms; *n* = 2313; *p* < 0.001, *U*-test; Fig. 3m). In neocortical recordings from opposite sides of the same dual-sided shank (thickness, 30 μm; Fig. 3n), we found a PYR-BIP pair with near-unity STG (0.961 spikes/spike) and a CCH peak at a sub-millisecond, unidirectional time lag (Fig. 3o). Thus, the vINT-BIP pairs and the dual-sided PYR-BIP analyses in the neocortex suggest that B-spikes correspond to recordings from non-somatic compartments.

### Positive phases of biphasic extracellular spikes correspond to return currents

**Fig. 4:**
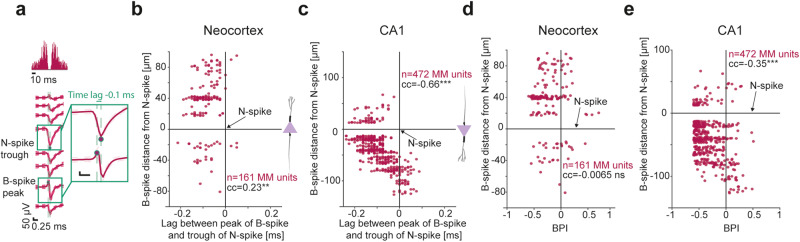
Positive phases of biphasic extracellular spikes correspond to return currents. **a** Computation of B-spike peak lag relative to the trough of the N-spike in the same MM unit. **b**, **c** Distance of B-spike from N-spike vs. lag of B-spike peak from N-spike trough. Distances are positive when the B-spike is closer to the surface of the brain. Cartoons illustrate the orientation of PYR soma, dendritic tree, and axon. Here and in (**c**), cc, rank correlation coefficient; **/***p < 0.01/p < 0.001, permutation test. The negative correlation in the neocortex (and the positive correlation in CA1) are consistent with the B-spike peaks near the soma and most proximal dendrites representing return currents during AIS spikes, and B-spike peaks near more distal dendrites representing return currents during somatic spikes. **d**, **e** Biphasic index (BPI) vs. distance between B- and N-spikes. The increasingly-positive BPIs farther from the N-spike (significant in CA1) are consistent with return currents forming a wavefront that propagates in space before the N-spike.

The analysis of same-unit B- to N-spike time lag depended on the B-spike trough (Fig. 2j; Fig. 3j, k). Because by definition the peak of a B-spike occurs before the trough, we also measured the time lag of the B-spike peak relative to the same-unit N-spike trough (Fig. 4a). In neocortex, the median time lag was -88 μs (95% confidence limits: [-100 -75] μs; *n* = 161 neocortical MM units; *p* < 0.001, Wilcoxon’s test; Supplementary Fig. 5a). Similar results were observed in CA1 (-100 [-113 -88] μs; *n* = 472; *p* < 0.001; Supplementary Fig. 5b). Because N-spikes, B-spikes, and P-spikes form a continuum (Supplementary Fig. 2c–f), a dichotomy between near-somatic P-spikes representing dendritic return currents and near-somatic B-spikes representing axonal potentials may be an oversimplification.

To determine the compartmental origin of the positive phase of B-spikes using blind extracellular recordings, we took advantage of the opposite axo-dendritic orientation of neocortical and CA1 PYRs (cartoons in Fig. 4b, c). Given the ripple-based physiological anchor in CA1 and the correspondence of N-spikes to somatic potentials, we tested where B-spikes appear in relation to N-spikes. We hypothesized that if there is a range of compartmental origins, either dendrites or axons, the time lag between B-spike peak and N-spike trough will depend on the distance from the N-spike. In addition, the correlation will be opposite in neocortex and CA1, mimicking the reversed axo-dendritic orientation of PYR in the two regions. Consistent with the predictions, in neocortex the cc was 0.23 (*n* = 161 MM units with B-spikes; *p* = 0.005, permutation test; Fig. 4b), whereas in CA1 the cc was -0.66 (*n* = 472; *p* < 0.001; Fig. 4c). Negative correlation was also observed in CA1 when the distance was measured from the B-spike to the center of stratum (str.) pyramidale (-0.36; *p* < 0.001; Supplementary Fig. 5c). Thus, the time lag vs. distance analyses indicate that B-spike peaks may either precede or be synchronized with same-unit N-spike troughs, suggesting that the B-spike peaks represent return currents.

For a spike generated at the axon initial segment, voltage mediated return currents are expected at the proximal dendrites and the axon^15,32,51^. However, once the spike begins propagating along the axon, return currents corresponding to the propagating spike are expected to be diminished at the side proximal to the soma due to refractoriness of voltage dependent channels. In turn, the axonal return currents will be increasingly asymmetrical in space and time as the distance increases. Measuring the BPI of B-spikes in MM units as a function of the distance from the N-spike, we found that in neocortex the cc was -0.0065 (*n* = 161 MM units; *p* = 0.56, permutation test; Fig. 4d), and in CA1 the cc was -0.35 (*p* < 0.001; Fig. 4e and Supplementary Fig. 5d–f). The polarity vs. distance results in CA1 suggest that spikes propagating along the unmyelinated part of the axon in close proximity with the N-spike exhibit an increasingly larger positive phase.

### BIPs and Punits exhibit place fields and phase precession

**Fig. 5:**
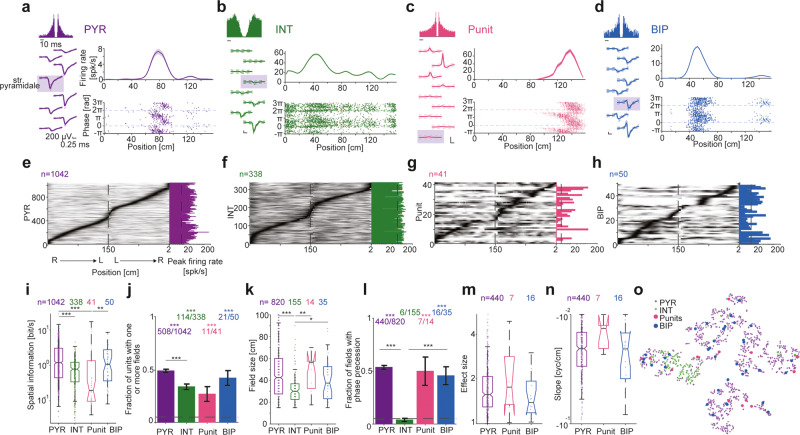
BIPs and Punits exhibit place fields and phase precession. **a**–**d** Units recorded in CA1 as mice ran back and forth on a 150 cm long linear track. In (**a**–**d**): Left, Wideband waveforms and ACH. Top, Firing rate as a function of position (mean ± SEM over 82/96/70/41 same-direction trials of PYR/INT/Punit/BIP). Bottom, Instantaneous theta phase of every spike. Phase 0 corresponds to theta peak. **e**–**h** Units of all four types exhibit increased firing rates at specific regions of the linear track. Every row represents a unit. Firing rates on right (R) to left (L) runs are concatenated with L to R runs and scaled to the 0 (white) to 1 (black) range. **i**–**k** Spatial rate coding of CA1 units. **i** Spatial information carried by the units. Top, number of units active and stable on the track. Here and in (**k**), */**/***p < 0.05/*p* < 0.01/*p* < 0.001, Kruskal–Wallis test, corrected for multiple comparisons. Box plot conventions are the same as in Fig. 2f. **j** Fraction of units with one or more place fields. Here and in (**l**), ***p < 0.001, Binomial test, comparing to chance level of 0.05; lined ***p < 0.001, *G*-test; error bars, SEM. **k** Place field sizes. **l**–**n** Spatial phase coding of CA1 units. **l** Fraction of fields with theta phase precession. **m** Phase precession effect sizes. **n** Phase precession slopes. **o** t-SNE projection of all fields. Sample size is the same as (**k**). Features includes are spatial information, field size, in-field gain, TPP effect size, and TPP slope.

Spatial coding by spiking neurons was studied extensively in the hippocampal formation^52^. There are differences between the coding properties of CA1 PYR and upstream regions. In particular, CA3 PYRs include a lower fraction of place cells, larger place fields, higher spatial information, and lower prevalence of phase precession^41^. To assess whether Punits and BIPs are part of the CA1 local circuit, we compared the spatial coding properties of PYRs, INTs, Punits, and BIPs recorded in CA1 while the mice ran back and forth along a 150 cm linear track. Units of all four types exhibited increased firing rates at specific parts of the track (Fig. 5a–d).

Focusing on active and stable units (Fig. 5e–h; Supplementary Table 4), the spatial information carried by BIPs was 1.07 [0.35 2.76] bit/s (*n* = 50 units), not consistently different from PYRs (1.18 [0.44 3.03] bit/s; *n* = 1042; *p* = 0.8; Kruskal–Wallis test; Fig. 5i). In contrast, Punits (0.19 [0.096 1.29] bit/s; *n* = 41), and INTs (0.78 [0.34 1.22] bit/s; *n* = 338) carried less spatial information than PYRs (*p* < 0.001). 508/1042 (49%) of the PYRs, 114/338 (34%) of the INTs, 11/41 (27%) of the Punits, and 21/50 (42%) of the BIPs had one or more place fields (Fig. 5j). On average, BIPs had 1.67 fields/unit, not consistently different from PYRs (1.61; *p* = 0.91, *G*-test; Supplementary Fig. 6a). In-field gain was 9.5 [1.1 239.5] for BIPs, not consistently different from PYRs (5 [1.9 27.6]; *p* = 0.92; Kruskal–Wallis test; Supplementary Fig. 6b). Field sizes of BIPs were 37.5 [22.5 52.5] cm (*n* = 35 fields), not consistently different from PYR fields (42.5 [27.5 60] cm; n = 820; *p* = 0.56), or from Punits fields (56.3 [30 57.5] cm; *n* = 14; *p* = 0.78; Kruskal–Wallis test; Fig. 5k). BIPs had larger field sizes than INT (30 [22.5 36.5] cm; *n* = 155; *p* = 0.018). Thus, in CA1, the spatial rate coding properties of BIPs are different from INTs but not distinct from PYRs.

As the animal advances through a place field, spikes of CA1 PYRs occur at progressively earlier theta phases, exhibiting theta phase precession^13,53^. Precession was more prevalent among PYR fields (440/820, 53.7%) compared to INT fields (6/155, 3.9%; *p* < 0.001), but not when compared with BIP (16/35, 45.7%; *p* = 0.6) or Punit fields (7/14, 50%; *p* = 0.88, *G*-test; Fig. 5l). Among phase precessing PYR fields, precession effect size was 1.56 [1.29 2.07] (*n* = 440), not consistently different from BIP (1.38 [1.17 1.8]; *n* = 16; *p* = 0.56) or Punit fields (1.76 [1 3.23]; *n* = 7; *p* = 0.94, Kruskal–Wallis test; Fig. 5m). Finally, the slope of phase precessing PYR fields was -0.02 [-0.031 -0.016] cyc/cm, not consistently different from BIP (-0.02 [-0.042 -0.019] cyc/cm; *p* = 0.96) or Punit fields (-0.014 [-0.024 -0.013] cyc/cm; *p* = 0.27, Kruskal–Wallis test; Fig. 5n). Thus, spatial phase coding of Punits and BIPs are not distinct from CA1 PYRs.

A t-SNE projection^54^ based on five spatial coding features (spatial information, field size, in-field gain, precession effect size, and precession slope) showed that BIP and Punit fields are interspersed within PYR fields (Fig. 5o). In summary, the similarity between the spatial coding properties of PYRs, Punits, and BIPs is consistent with non-negative potentials representing cellular compartments of local circuit CA1 PYRs.

### In CA1, Punits and BIPs occur outside str. pyramidale

**Fig. 6:**
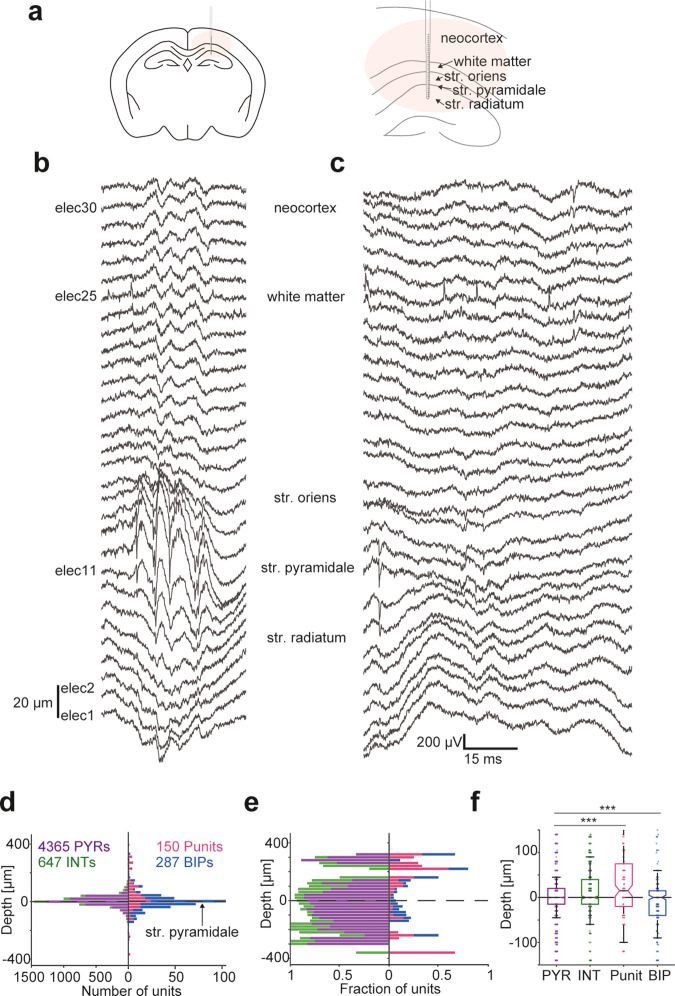
In CA1, Punits and BIPs occur outside str. pyramidale. **a** Linear recording array in the neocortex and CA1. **b** Wideband traces recorded by the 32-electrode array in a freely-moving mouse. Vertical inter-electrode spacing is 20 µm. A ripple event peaks at elec11, corresponding to the center of str. pyramidale. **c** Traces from the same session showing multiple spike events, including a PYR in str. pyramidale (elec8-11), a SM Punit in the white matter (elec25), and a neocortical PYR (elec30-31). **d** Depth distribution of PYRs, INTs, Punits, and BIPs in CA1. Zero depth corresponds to the center of CA1 str. pyramidale. Bin size is 20 µm. Here and in (**e**, **f**), horizontal dashed lines indicate the center of the layer. **e** Same as (**d**), with the absolute numbers scaled to fractions at every depth. **f** The depth distribution of non-negative units is distinct from the depth distribution of PYRs in CA1. ***p < 0.001, Kolmogorov–Smirnov test. Box plot conventions are the same as in Fig. 2f.

In simultaneous recordings of neocortex and CA1 (Fig. 6a), SM Punits were observed 200–300 μm above CA1 str. pyramidale, corresponding to the white matter in the corpus callosum (Fig. 6b, c). We determined which electrodes were located at the center of str. pyramidale based on spontaneous high frequency oscillation ripple events (Fig. 6b). In probes that span a vertical range of 300–620 μm, Punits appeared mainly in the white matter, 210 [-40 260] μm (*n* = 30) above str. pyramidale. Most white matter Punits were SM units (29/30, 97%). Thus, SM non-negative units may correspond to return currents close to the soma (Fig. 1) and to axonal potentials in myelinated fibers.

In CA1 hippocampal region, str. pyramidale includes PYR somata, whereas str. oriens and str. radiatum include non-somatic compartments of CA1 PYR and presynaptic terminals. Close to str. pyramidale, the fraction of non-negative units was lowest at the center of the layer and higher in the flanking strata (Fig. 6d, e). The depth distribution of SM Punits (*n* = 150) and SM BIP (*n* = 287) differed from the depth distribution of SM PYRs (*n* = 4365; *p* < 0.001, Kolmogorov–Smirnov test; Fig. 6f). Whereas the median [IQR] depth of PYRs was 0 [-15 20] μm, Punits were closer to str. oriens (15 [-20 75] μm; *p* < 0.001, *U*-test). Furthermore, the dispersion of Punit depths (SD, 115 μm) was higher than of PYRs (50 μm; *p* = 0.004, permutation test). BIPs were recorded at depths of 0 [-40 15] μm, closer to str. radiatum compared to PYRs (*p* < 0.001, *U*-test). Thus, the CA1 sub-layer organization of Punits and BIPs differs from PYRs, implying that the origin of Punits and BIPs is not somatic.

### Punits and BIPs precede PYRs and INTs in ripple lock in CA1

**Fig. 7:**
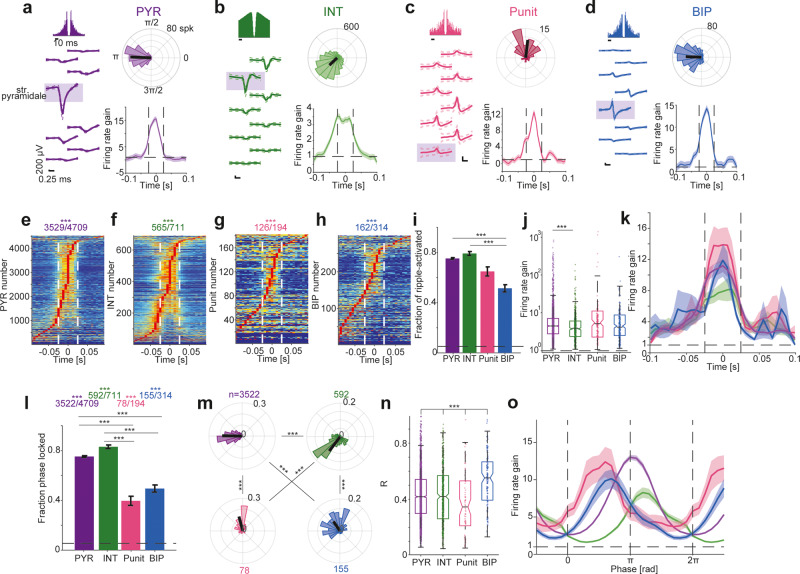
Punits and BIPs precede PYRs and INTs in ripple lock in CA1. **a**–**d** Units with non-negative waveforms exhibit time-locking to ripple events. Example ripple-locked CA1 PYR, INT, Punit, and BIP. Left, Wideband spike waveforms and ACHs. Top right, Ripple phase during every spike, binned into 20 equal-sized bins. Black line represents the mean phase and the resultant length. Bottom right, Firing rate gain as a function of time in ripple. Here and in (**e**–**h**), vertical dashed lines indicate mean ripple time range. **e**–**h** Punits and BIPs exhibit increased firing rates during ripple events. Ripple-triggered firing rate histograms of all CA1 units. For presentation purposes, firing rates are scaled to the 0 (blue) to 1 (red) range. ***p < 0.001, Binomial test comparing to chance level, 0.05. **i** The fract**i**on of BIPs with firing rate gain above 1 during ripple events. Here and in (**l**), lined ***p < 0.001, *G*-test, corrected for multiple comparisons; error bars, SEM. Vertical dashed lines indicate chance level, *α* = 0.05. **j** Firing rate gain during ripples. Here and in (**n**), ***p < 0.001, Kruskal–Wallis test. Box plot conventions are the same as in Fig. 2f. **k** Gain as a function of time in ripple. Here and in (**o**), shading shows SEM. **l**–**o** Phase-locked Punits and BIPs precede PYRs and INTs in ripple events. **l** The fraction of phase-locked units. **m** Punits and BIPs spike earlier on the ripple cycle compared to PYRs and INTs. ***p < 0.001, Wheeler–Watson test. **n** Resultant lengths of ripple phase, indicating lock strength. **o** Gain as a function of phase in ripple.

As an independent assessment of the possibility that Punits and BIPs may represent non-somatic potentials of local circuit neurons in CA1, we analyzed spiking activity during ripple oscillations. Akin to PYR and INT, firing rates of non-negative units increased during ripples (Fig. 7a–d). Units of all four populations exhibited consistent (*p* < 0.05, Poisson test) firing rate increases during ripple events (*p* < 0.001, Binomial test; Fig. 7e–h). The fraction of BIP with increased ripple firing rates was 162/314 (51.6%), lower than PYRs (3529/4709, 75%; *p* < 0.001) and INT (565/711, 79.5%; *p* < 0.001), but not consistently different from Punits (126/194, 65%; *p* = 0.75, *G*-test; Fig. 7i). Of the units with increased ripple firing rates, the gain of Punits (4.8 [2.5 8.6], *n* = 126) and BIP (4.62 [2.12 11.28], *n* = 162) was not consistently different from PYRs (4.47 [2.77 7.13], *n* = 3529; *p* = 0.96 and *p* = 0.89, respectively; Kruskal–Wallis test; Fig. 7j). Thus, the firing rates of Punits and BIPs are temporally modulated by ripples (Fig. 7k), consistent with spiking of CA1 local circuit neurons.

The fraction of phase-locked Punits (78/194, 40.2%) and BIPs (155/314, 49.4%) was above chance (0.05; *p* < 0.001, Binomial test; Fig. 7l) but lower than phase-locked PYRs (3522/4709, 74.8%) and INTs (592/711, 83.3%; *p* < 0.001, *G*-test; Fig. 7l). Due to the curvature of dorsal CA1, a parallel multi-shank electrode array inserted perpendicularly to CA1 at the point of penetration may record only non-somatic compartments of some neurons (Fig. 7c, d). Superficial units (closer to str. radiatum) spike earlier in the ripple cycle than deep units (closer to str. oriens^55^). Every population fired at a different phase: earliest were Punits (0.53π [0.46π 0.87π]; *n* = 78), followed by BIPs (0.65π [0.46π 1.02π]; *n* = 155), PYRs (1.01π [0.89π 1.11π]; *n* = 3522) and finally INTs (1.25π [1.11π 1.48π]; *n* = 592; *p* < 0.001, Wheeler–Watson test; Fig. 7m). The length R of the circular resultant vector ranges [0,1] and is a straightforward measure of phase-locking magnitude. The within-unit resultant length R of Punits (0.35 [0.14 0.53]) was lower than of PYRs (0.42 [0.3 0.54]) and INT (0.42 [0.26 0.57]), and highest for BIP (0.55 [0.39 0.67]; *p* < 0.001, Kruskal–Wallis test; Fig. 7n). Thus, Punits and BIPs are phase-locked to high frequency ripples but spike earlier than PYRs in the ripple cycle (Fig. 7o), suggesting that Punits and BIPs correspond to non-somatic compartments of PYRs that reside in superficial CA1 layers.

## Discussion

Using high-density extracellular recordings from freely-moving mice, we investigated the possible origin of non-negative extracellular waveforms. We found that P-spikes and positive phases of B-spikes are most consistent with return currents, and that isolated P- or B-spikes are most consistent with axonal potentials. In hippocampal region CA1, Punits and BIPs carry spatial information, exhibit phase precession, and are phase-locked to ripples, akin to local circuit PYRs.

It was suggested that P-spikes in vitro^17^ and B-spikes in vivo^34,35^ correspond to active backpropagation of somatic spikes into dendrites^43^. Previously-reported non-negative spikes were seen far from the somatic potentials^36^, whereas most of our P- and B-spikes were in close proximity to the N-spikes. Backpropagating spikes are wider than somatic signals^40,43^. Because the P- and B-spikes in the present dataset are narrower than N-spikes, the non-negative spikes are unlikely to be produced by backpropagation.

Return currents represent the instantaneous outward flow of positive ions during an action potential, balancing charges and maintaining a steady membrane potential. P-spikes in vitro^19,20^ and in silico^15,16^ were suggested to correspond to return currents. Up to charge movement times, return currents should occur at the same time as spike generation at the AIS^56^. Therefore, spikes representing return currents are expected to occur in sync or slightly before the somatic spike. We found that P-spikes occur in synchrony with same-unit N-spikes. We also found that B-spike troughs occur after same-unit N-spikes, and that the positive phase of B-spikes occurs before the same-unit N-spikes. These observations suggest that when a spike is generated at the axon initial segment, the balancing return currents are evident as the positive B-spike phase in the extracellular record. Then, the AIS spike propagates to the soma and is evident as an N-spike, resulting in a slight delay of the N-spike relative to the positive phase of the B-spike.

B-spikes in vitro^7,23,25–27^, in silico^9,15,32^, and in vivo^21,24,28^, and P-spikes^21,22^ were suggested to correspond to axonal potentials. The positive peak of a B-spike far from the N-spike can be viewed as a local return current. As an AIS-generated spike propagates along the axon, the return currents propagate as well. For an unmyelinated juxta-somatic axon, the return currents of the propagating spike are expected distally because the sodium channels proximal to the soma are refractory. When recorded at a single point in space near the axon over time, the wavefront of the positive peak occurs before the negative spike, yielding a peak-then-trough biphasic spike.

Sodium spikes can be generated not only in the soma/AIS but also in the dendrites^57–59^. In vitro^18,31^ and in silico^32,33^, B-spikes were suggested to be generated in the dendrites by forward propagation of dendritic sodium spikes. In MM units, P-spikes were in sync with N-spikes, and the trough of the B-spikes occurred after the N-spike trough. Therefore, it is unlikely that P- and B-spikes were created by forward propagation of dendritic sodium spikes. Dendrites can also generate calcium spikes^60,61^. In a dendritic calcium spike, P- and B-spikes are expected to appear before the somatic spike, but calcium spikes are much wider. Thus, the most likely compartmental counterparts of MM P- and B-spikes are dendritic return currents and axonal potentials.

Isolated P- and B-spikes both corresponded to axonal spikes. Return currents in myelinated axons can flow via nodes of Ranvier, manifesting as isolated positive phase potentials that correspond to SM Punits. It is possible that the difference between the P- and B-spike waveforms originates from difference in the myelin sheath. In neocortical PYRs, the axon becomes myelinated 35–50 μm after the AIS^62^, 75–90 μm from the soma^63^. INT axons myelinate closer to the soma, 20–50 μm^64^. Axons of CA1 PYRs myelinate even closer, within 10–20 μm^65^. The early myelination for neocortical INTs and CA1 PYRs may explain the scarcity of MM INTs and the lower prevalence of MM units in CA1.

The short time lags of excitatory vINT-BIP pairs and the high-fidelity PYR-BIP transmission may suggest that B-spikes are generated by inter-neuronal gap junctions^66–68^. However, gap junction prevalence among neocortical PYRs is low^68^, inconsistent with the observation of neocortical MM PYRs with non-negative spikes. Moreover, neurons coupled by gap junctions typically yield CCHs with two-sided peaks^67,69^, whereas the CCHs of the excitatory vINT-BIP and PYR-BIP pairs are unidirectional. Therefore, it is unlikely that “excitatory” connections to BIP were caused by gap junctions.

A possible source of SM Punits is sporadic juxtacellular recording, established by electrode adhesion to the membrane^70^. However, the sizes of the electrodes employed (10 × 15 μm) and the diameter of a PYR soma (10–15 μm^71^) are similar. Furthermore, SM Punit waveforms were recorded together with waveforms of other units by the same electrode. Thus, non-negative extracellular waveforms are unlikely to be due to juxtacellular recordings.

In principle, P-spikes can be generated by somatic potassium action potentials. Potassium spikes were previously observed as slow “hyperpolarizing” responses in invertebrate muscles^72,73^, and may occur in neurons when extracellular potassium concentrations are very high or when potassium channels are inactivated^74^. However, the presently-reported P-spikes occur together with N-spikes and are much sharper than previously-reported potassium spikes.

Non-negative spikes comprise about 10% of the waveforms, but past studies rarely reported non-negative spikes. The discrepancy may be due to low-density recordings, limited detection parameters, and spike sorting bias. To minimize waveform distortion due to filtering^75,76^, we employed a very low high-pass frequency (0.1 Hz). We expect the application of similar signal processing techniques to increase the prevalence of non-negative spikes reported in the future.

The fraction of P-spikes was higher in neocortex compared with CA1. The lower prevalence of P-spike units in CA1 may be due to a shadowing effect, caused by the high density of PYRs in str. pyramidale. Due to the layered organization of hippocampal region CA1, the shanks of a probe implanted vertically in the dorsal hippocampus and centered on str. pyramidale are parallel to the axo-dendritic tree of the PYRs. Therefore, B- and N-spikes of CA1 PYRs may be recorded simultaneously, yielding MM PYRs, consistent with the higher probability of observing MM units with B-spikes in CA1 compared with the neocortex. In contrast, SM BIP and Punits are likely to represent compartments of units with unrecorded somata. In CA1, isolated compartmental recordings may represent cell bodies outside str. pyramidale, or PYRs whose axo-dendritic axes are not parallel to the probe due to the curvature of the hippocampus. Indeed, modeling^77^ showed that for a polarized multi-compartmental neuron, the relative orientation of the electrode array and the axo-dendritic axis impacts the recorded waveforms.

It is a-priori possible that some of the CA1 Punits and BIPs represent non-somatic (e.g., axonal) compartments of CA3 neurons projecting to CA1. Place coding in CA3 PYRs includes lower fraction of place cells, larger place fields, higher spatial information, and lower phase precession prevalence^41^. However, the prevalence and properties of place coding by SM CA1 BIPs and Punits were not consistently different from CA1 PYR place coding. Thus, the properties of Punits and BIPs recorded in CA1 are inconsistent with CA3 place coding. Because studies of hippocampal place coding typically analyze spike timing but spike waveforms are only rarely shown, it is unknown whether previous studies considered Punits and BIPs as place cells.

While the present dataset yielded many non-negative waveforms, the structure of the multi-shank probes (vertical span of 100-200 μm) and electrode size are optimized for high-density somatic recordings. The limited vertical span precludes monitoring distant potentials, and the prevalence of MM units may be even higher with higher-span probes^2^. A second interpretational limitation is related to the employed electrode size. A large electrode averages potentials from multiple small sources: the diameter of a neocortical PYR axon is 0.2-0.5 μm^78^, and the diameter of an apical proximal dendrite is 1-4 μm^79^. Smaller electrodes (e.g., 7 μm or smaller^80^) may capture more potentials generated by non-somatic compartments, with the possible caveat of higher impedance and thermal noise. A third limitation is due to the fact that we did not rely on histological confirmation, but rather employed an established physiological anchor for identifying the CA1 pyramidal cell layer, high frequency ripples.

Punits and BIPs were most consistent with return currents and axonal spikes, but all findings were correlative and we did not demonstrate causality. A causal link may be achieved using compartmental blockade. During recordings from the CA1 feedforward network, a specific blocker for axonal sodium channels (NaV1.2^81,82^) may be applied locally. We predict that MM PYRs with B-spikes to be recorded before and during the blockade will turn to SM PYRs with only N-spikes during the blockade, and that BIPs will disappear. Alternatively, if sodium channels in proximal dendrites are blocked (NaV1.6^83^), the N-spike to P-spike distance is expected to increase in MM PYRs with P-spikes.

Punits and BIPs may be further classified into different types. For instance, the present dataset already includes several relatively wide P-spikes, which may correspond to backpropagating spikes^34,35^. Because both PYR and INT can exhibit B-spikes, classification of single-modal units with non-negative spikes into distinct cell types^84^ may be possible. Furthermore, classifiers agnostic to waveform shape or polarity^85,86^ may associate non-negative spikes with specific cell types. The full dataset and relevant code for categorizing waveforms and classifying units are available.

The analysis of P- and B-spikes may have direct implications for studying neural diseases. Presently, measurements of axonal propagation velocity are carried out ex vivo^49,64,87–89^ or under anesthesia^90^. Future studies may measure axonal propagation velocity in the intact brain directly by computing time lags between N- and B-spikes in MM units with B-spikes. The approach may enable studying neurological disorders associated with axonal pathology, including Alzheimer’s disease^91^ and multiple sclerosis^92,93^.

## Methods

### Experimental animals

A total of 17 freely-moving mice were used in this study (Supplementary Table 1). 16 of the mice were males and one was female. Four of the mice were first-generation hybrids (FVB/NJ x C57BL/6 J)F1, and 13 were on a C57BL background^94^. The mice aged 8–32 weeks (median, 16.5 weeks) at the time of implantation. Animals were healthy, were not involved in previous procedures, and weighed 24.2–35.9 g (median, 30.5 g) at the time of implantation. Mice were single housed to prevent damage to the implanted apparatus. All animal handling procedures were in accordance with Directive 2010/63/EU of the European Parliament, complied with Israeli Animal Welfare Law (1994), and approved by Tel Aviv University Institutional Animal Care and Use Committee (IACUC #01-16-051).

### Probes and surgery

Every animal was implanted with a multi-shank silicon probe attached to a movable microdrive^95^. The probes used were Stark64 (Diagnostic Biochips; six mice), Buzaski32 (NeuroNexus; five mice), Linear32 (A1x32-Edge-10mm-20-177, NeuroNexus; four mice) and Dual-sided64 (DS64, Diagnostic Biochips; two mice). The Stark64 probe consists of six shanks, spaced horizontally 200 μm apart, with each shank consisting of 10–11 recording sites, spaced vertically 15 μm apart. The Buzaski32 probe consists of four shanks, spaced horizontally 200 μm apart, with each shank consisting of eight recording sites, spaced vertically 20 μm apart. The Linear32 probe consists of one shank, with 32 recording sites, spaced vertically 20 μm apart. The DS64 probe consists of two 30 μm thick dual-sided shanks, spaced horizontally 250 μm apart, with each shank consisting of 16 channels on each side (front and back), spaced vertically 20 μm apart. Probes were implanted in the neocortex above the right hippocampus (PA/LM, 1.6/1.1 mm; 45° angle to the midline) under isoflurane (1%) anesthesia^96^.

### Recording sessions

Recording sessions lasted 4.3 [3.4 5.7] h (median [IQR] of 197 sessions). After every session, the probe was translated vertically downwards by no more than 70 μm. All hippocampal recordings were from the CA1 layer, recognized by the appearance of multiple high-amplitude units and iso-potential spontaneous ripple events. In every session, neural activity was recorded while the animal was in the home cage. Animals were equipped with a 3-axis accelerometer (ADXL-335, Analog Devices) for monitoring head movements.

### Linear track sessions

During every session that involved running on the linear track (Supplementary Table 4), neural activity was first recorded while the animal was in the home cage for at least 40 min. The animal was then placed on a 150 cm linear track that extended between two 10 × 10 cm square platforms. Each platform included a water delivery port. Mice were under water restriction and were trained to repeatedly traverse the track for a water reward of 3–10 μl. Head position and orientation were tracked in real time using two head-mounted LEDs, a machine vision camera (ace 1300–1200 uc, Basler), and a dedicated system (Spotter^97^). Four mice ran a total of 75 sessions that included 168 [130 212] one-direction trials over 50–90 min. Trials with a mean running speed below 10 cm/s were excluded from analyses.

### Spike detection and sorting

Neural activity was filtered, amplified, multiplexed, and digitized on the headstage (0.1–7500 Hz, x192; 16 bits, 20 kHz; RHD2132 or RHD2164, Intan Technologies), and then recorded by an RHD2000 evaluation board (Intan Technologies). Offline, spikes were detected, detrended (Supplementary Fig. 2b), and sorted into single units automatically using KlustaKwik3^98^ for shanks with up to 11 sites/shank, or KiloSort2^99^ for shanks with 16-32 channels. Automatic spike sorting was followed by manual adjustment of the clusters. Only well-isolated units were used for further analyses (amplitude >40 μV; L-ratio <0.05; inter-spike interval index <0.2^50^).

### Categorization of spike waveforms

For every well-isolated unit, the mean waveform recorded on every electrode was categorized as an N-spike, P-spike, B-spike, or left uncategorized. Waveform categorization proceeded as follows. First, the waveforms of all spikes were averaged over all spikes, and the mean and SD were denoted. The mean of the first 0.15 ms (3 samples) was subtracted from the mean waveform, and then the mean and the SD waveform were upsampled four-fold in time using cubic spline interpolation, yielding two 128-sample vectors. Second, all local extrema of the mean upsampled vector were detected. The local extremum with maximal value *p* was denoted as the peak, and the local extremum with the minimal value *n* was denoted as the trough. For every waveform, a bipolar index was computed, defined as

1 $${{{{{\rm{BPI}}}}}}=({{{{{\rm{p}}}}}}-{{{{{\rm{|n|}}}}}})/({{{{{\rm{p}}}}}}+{{{{{\rm{|n|}}}}}}).$$

The BPI ranges -1 to 1, taking a value of zero when the peak and trough have the same absolute value (Supplementary Fig. 2c–f).

B-spikes: If the peak preceded the trough, the peak was larger than 1.25 SDs at the peak and the trough was larger than one SD at the trough, and -0.6 < BPI < 0.8, the waveform was categorized as a B-spike.

P-spikes: A waveform not categorized as a B-spike that had a peak larger than the trough *p* > |*n* | , and the peak was larger than 1.75 SDs at the peak, was categorized as a P-spike.

N-spikes: A waveform not categorized as a B-spike that had a trough larger than the peak |*n* | >*p*, and an absolute value of the trough larger than 1.75 SDs at the trough, was categorized as an N-spike.

All other waveforms were uncategorized. Overall, we categorized 2112 spikes as B-spikes, 1434 as P-spikes, and 37255 as N-spikes. 60383 spikes were not categorized. A median [IQR] of 4 [3 6] spikes were categorized per unit. We used ~2 SDs since in a Gaussian distribution, 1.96 SDs correspond exactly to 0.05, the significance threshold (α). We verified that the categorization of waveforms was not sensitive to the specific parameter values noted above. In particular, when using a symmetric definition for B-spikes (one SD at the peak and one SD at the trough) and equivalent definitions for P-spikes and N-spikes (two SDs at the peak and trough, respectively), the number of B-/P-/N-spikes were 3123, 1097, and 32652.

### Classification of units

For every unit, the waveform with the maximal magnitude, defined as the difference between the maximal value and the minimal value, was denoted the main channel. Every unit was then classified according to the spike category of the waveform in the main channel. Thus, units with a B-spike in the main channel were classified as B-spike units (BIPs); units with a P-spike in the main channel were classified as P-spike units (Punits); and units with an N-spike in the main channel were classified as N-spike units.

In addition, every unit was classified as a single-modal (SM) or a multi-modal (MM) unit. If all categorized constituent spikes had the same categorization (e.g., all were N-spikes), the unit was classified as SM. All other units were classified as MM.

Finally, negative-spike units were classified into putative pyramidal cells (PYRs) or PV-like interneurons (INTs) using a Gaussian mixture model^42^ (Supplementary Fig. 1a). Non-negative spikes did not adhere to the mixture model (Supplementary Fig. 1b).

A total of 9160 units were recorded from the 17 mice during 197 sessions (Supplementary Table 1). Of these, 6959 (76%) were PYRs, 1334 (14.6%) were INTs, 378 (4.1%) were Punits, and 489 (5.3%) were BIPs. As for the categorization of spikes, we verified that the classification of units was not sensitive to the specific parameter values. In particular, when using the alternate set of parameter values, the number of PYR/INT/Punits/BIP were 6879/1323/375/571. A total of 3189 units were recorded from the neocortex during 78 sessions (Supplementary Table 3), and 5971 units were recorded from CA1 during 126 sessions (Supplementary Table 2).

### Determining monosynaptic connectivity

To determine whether a monosynaptic connection may exist between units, count cross-correlation histograms (CCHs; 0.1 ms bins) were constructed for putative pre- and postsynaptic spike train pairs^100^. The spike transmission curve was estimated by the difference between the deconvolved CCH and the baseline, determined by hollowed median filtering of the count CCH, scaled to spikes/s. The spike transmission gain (STG) was defined as the area under the peak in the monosynaptic temporal region of interest (ROI; 0<t ≤ 5 ms), extended until the causal zero-crossing points. The STG estimates the number of postsynaptic spikes generated following a presynaptic spike. Units that participated as a reference in a CCH that exhibited a consistent peak (*p* < 0.001, Bonferroni-corrected Poisson test on the deconvolved count CCH compared to baseline) in the monosynaptic ROI were defined as presynaptic excitatory cells. Units that participated as a reference in a CCH that exhibited a consistent trough in the monosynaptic ROI were defined as presynaptic inhibitory cells.

### Identification of place fields

All analyses were performed only on active and stable units^101^. Active units fired a minimum of 5 spikes in at least one 2.5 cm spatial bin on the linear track, pooled over all trials. To determine whether a unit was stable, all rank correlation coefficients between the firing rate maps of same-direction trial pairs were computed. Statistical significance was determined by the geometric mean p-values derived from a permutation test over all pairs in both running directions. Units with a p-value below chance were considered stable. Overall, 1471/4515 (32.6%) of the recorded units were active and stable (Supplementary Table 4).

For every active and stable unit, place fields were defined in regions of space in which the on-track firing rate deviated from chance level, determined recursively based on a Poisson distribution^101^. Place fields that contained fewer than 30 spikes were omitted from further analyses. Precession effect size was quantified as the ratio between the fit of spikes to a circular-linear model divided by the median of 300 model fits to randomly permuted phase/position pairs^94^.

### Analysis of ripples

High frequency ripple oscillations in CA1 were detected independently at each electrode^55^. The wideband signal was bandpass-filtered (80–250 Hz; difference-of-Gaussians, DOG; zero-lag, linear phase FIR), and instantaneous power was computed by clipping values above five SDs, rectifying, and low-pass filtering. The mean and SD were computed from the power of the clipped signal during non-theta immobility. Events for which the power of the unclipped signal exceeded five SDs from the mean were detected, expanded until the power fell below two SDs to define event edges, and aligned by the trough closest to the peak power. The center of the CA1 pyramidal cell layer was determined for each shank by the maximal ripple amplitude, and region assignment was done per unit. Subsequent analyses were based on ripples detected at the center of the layer. For every unit, ripple gain was defined by the ratio of the firing rate during spontaneous ripple events and the baseline firing rate of that unit, as measured during non-theta immobility. The instantaneous phase of the DOG-filtered signal was derived from its Hilbert transform, and spikes that occurred during a ripple event were assigned the phase (0-2π) at the time of firing.

### Statistics and reproducibility

In all statistical tests a significance threshold of *α* = 0.05 was used. An exception was the threshold used for determining whether two units exhibit monosynaptic connectivity (*α* = 0.001). In all cases, nonparametric testing was used. An exception was to determine ripple phase locking (Rayleigh test). All statistical details (*n*, median, IQR, range, confidence limits, mean, SEM) can be found in the main text, figures, figure legends, and supplementary tables. To estimate whether fractions were larger or smaller than expected by chance, an exact one-tailed Binomial test was used. Differences in the proportions of two categorical variables were tested with a likelihood-ratio (*G*-) test (two-tailed). Bonferroni’s correction was employed in cases of *G*-test multiple comparisons. Differences between two group medians were tested with either Mann-Whitney’s *U*-test (unpaired samples) or Wilcoxon’s paired signed-rank test (two-tailed). Differences between medians of four groups were tested with Kruskal–Wallis test, and corrected for multiple comparisons using Tukey’s procedure. Wilcoxon’s signed-rank test was employed to determine whether a group median is distinct from a predetermined value (two tailed). Association between parameters was quantified using Spearman’s rank correlation and tested with a permutation test. The statistical significance of unimodal phase locking was tested using Rayleigh’s likelihood-ratio test of uniformity. Differences between the circular medians of two groups were tested with Wheeler–Watson’s nonparametric two-sample test (two-tailed). For all figures, *p < 0.05; **p < 0.01; ***p < 0.001.

### Reporting summary

Further information on research design is available in the Nature Portfolio Reporting Summary linked to this article.

### Supplementary information

Supplementary Information

Description of Additional Supplementary Files

Supplementary Data 1

Reporting Summary

## Data Availability

The source data behind the graphs in the paper are available in Supplementary Data 1. The data used in this study are available from the corresponding author upon reasonable request. The waveform data of all 9160 units used in the study are hosted publicly at 10.5281/zenodo.8286842.

## Code availability

Code for implementing waveform categorization and unit classification, along with example spike data, are hosted publicly on GitHub, accessible via https://github.com/EranStarkLab/NonNegativeSpikes.
