# Supplementary material for: Positive and biphasic extracellular waveforms correspond to return currents and axonal spikes
Source: Commun Biol. 2023 Sep 18;6:950. doi: 10.1038/s42003-023-05328-6 (PMC10507124; doi:10.1038/s42003-023-05328-6)
Supplement: Supplementary file 1 — Supplementary Information [file 42003_2023_5328_MOESM1_ESM.pdf]

Supplementary information for

Positive and biphasic extracellular waveforms correspond to return currents and axonal spikes

Shirly Someck, Amir Levi, Hadas E. Sloin, Lidor Spivak, Roni Gattegno, Eran Stark

Correspondence: eranstark@sci.haifa.ac.il

Supplementary information

Supplementary Tables 1-4

Supplementary Figures 1-6

Supplementary Tables

Supplementary Table 1. Mice used for electrophysiological recordings

Animal ID	Sex	Age[1] [week]	Weight[1] [g]	Strain[2]	Probe	Sessions	Units[3]	Neocortex sessions	CA1 sessions	CA1 linear track sessions
mA234	M	16	30	HYB	Buzsaki32	40	2285	14	26	24
mB142	M	20	35.9	HYB	Buzsaki32	1	10	1	0	0
mC41	M	10	33.7	HYB	Stark64	37	2473	5	32	32
mDL5	M	32	30.9	C57	Buzsaki32	14	220	14	0	0
mDS1	M	14	25.7	C57	DS64[4]	21	861	4	17	5
mDS2	F	30	24.2	C57	DS64[4]	12	1546	9	3	0
mF105	M	26	32.5	HYB	Stark64	1	57	1	0	0
mF108	M	12	31.4	C57	Stark64	2	138	2	0	0
mF79	M	17	30.1	C57	Stark64	1	41	1	0	0
mF84	M	8	24.4	C57	Linear32	8	126	8	0	0
mF93	M	18	33.5	C57	Stark64	2	170	2	0	0
mK01	M	24	29.5	C57	Linear32	5	67	3	5	0
mO251	M	13	31.5	C57	Linear32	5	52	2	4	0
mP101	M	16	29.7	C57	Buzsaki32	6	231	1	5	0
mP20	M	20	31	C57	Stark64	4	165	4	0	0
mP23	M	14	31.2	C57	Buzsaki32	25	666	3	23	14
mV99	M	N/A	25.8	C57	Linear32	13	52	4	11	0
**Total**						**197**	**9160**	**78**	**126**	**75**

[1] At the time of implantation.

[2] HYB, hybrid mice. C57, mice on a C57BL background.

[3] Total number of well-isolated units recorded during every session.

[4] Dual-sided64 probe.

Supplementary Table 2. Units recorded from CA1

			MM					SM				
**Animal ID**	**Sessions**	**Units**	**Total**	**PYR**	**INT**	**Punit**	**BIP**	**Total**	**PYR**	**INT**	**Punit**	**BIP**
**mA234**	26	1699	162	108	0	0	54	1537	1276	191	35	35
**mC41**	32	2326	366	254	3	1	108	1960	1536	285	88	51
**mDS1**	17	803	63	58	0	0	5	740	645	74	18	3
**mDS2**	3	195	12	6	1	0	5	183	116	42	20	5
**mK01**	5	49	13	12	1	0	0	36	26	8	2	0
**mO251**	4	46	8	6	2	0	0	38	28	9	0	1
**mP101**	5	223	30	19	0	0	11	193	150	23	7	13
**mP23**	23	587	34	25	1	0	8	553	462	65	13	13
**mV99**	11	43	1	0	0	1	0	42	19	19	3	1
**Total**	**126**	**5971**	**689**	**488**	**8**	**2**	**191**	**5282**	**4258**	**716**	**186**	**122**

Supplementary Table 3. Units recorded from neocortex

			MM					SM				
**Animal ID**	**Sessions**	**Units**	**Total**	**PYR**	**INT**	**Punit**	**BIP**	**Total**	**PYR**	**INT**	**Punit**	**BIP**
**mA234**	14	586	37	15	4	2	16	549	360	137	35	17
**mB142**	1	10	2	1	0	0	1	8	5	1	1	1
**mC41**	5	147	3	0	0	0	3	144	101	12	22	9
**mDL5**	14	220	3	2	0	0	1	217	158	47	9	3
**mDS1**	4	58	1	1	0	0	0	57	27	21	5	4
**mDS2**	9	1351	179	126	11	5	37	1172	942	187	29	14
**mF105**	1	57	0	0	0	0	0	57	43	11	2	1
**mF108**	2	138	12	3	1	0	8	126	56	53	7	10
**mF79**	1	41	0	0	0	0	0	41	29	6	2	4
**mF84**	8	126	13	6	1	1	5	113	70	24	15	4
**mF93**	2	170	18	9	1	0	8	152	87	55	7	3
**mK01**	3	18	1	1	0	0	0	17	13	0	4	0
**mO251**	2	6	0	0	0	0	0	6	2	1	2	1
**mP101**	1	8	0	0	0	0	0	8	1	5	2	0
**mP20**	4	165	6	0	0	3	3	159	106	15	21	17
**mP23**	3	79	2	0	0	0	2	77	44	16	13	4
**mV99**	4	9	0	0	0	0	0	9	5	1	3	0
**Total**	**78**	**3189**	**277**	**164**	**18**	**11**	**84**	**2912**	**2049**	**592**	**179**	**92**

Supplementary Table 4. Units recorded from CA1 on the linear track

				MM					SM				
**Animal ID**	**Sessions**	**Units**	**Active units[1]**	**Total**	**PYR**	**INT**	**Punit**	**BIP**	**Total**	**PYR**	**INT**	**Punit**	**BIP**
**mA234**	24	1633	572	20	6	0	0	14	552	402	124	15	11
**mC41**	32	2326	737	42	26	1	0	15	695	485	175	25	10
**mDS1**	5	237	58	1	1	0	0	0	57	45	12	0	0
**mP23**	14	336	104	1	1	0	0	0	103	76	26	1	0
**Total**	**75**	**4515**	**1471**	**64**	**34**	**1**	**0**	**29**	**1407**	**1008**	**335**	**41**	**21**

[1] Active and stable on the linear track.

Supplementary figures

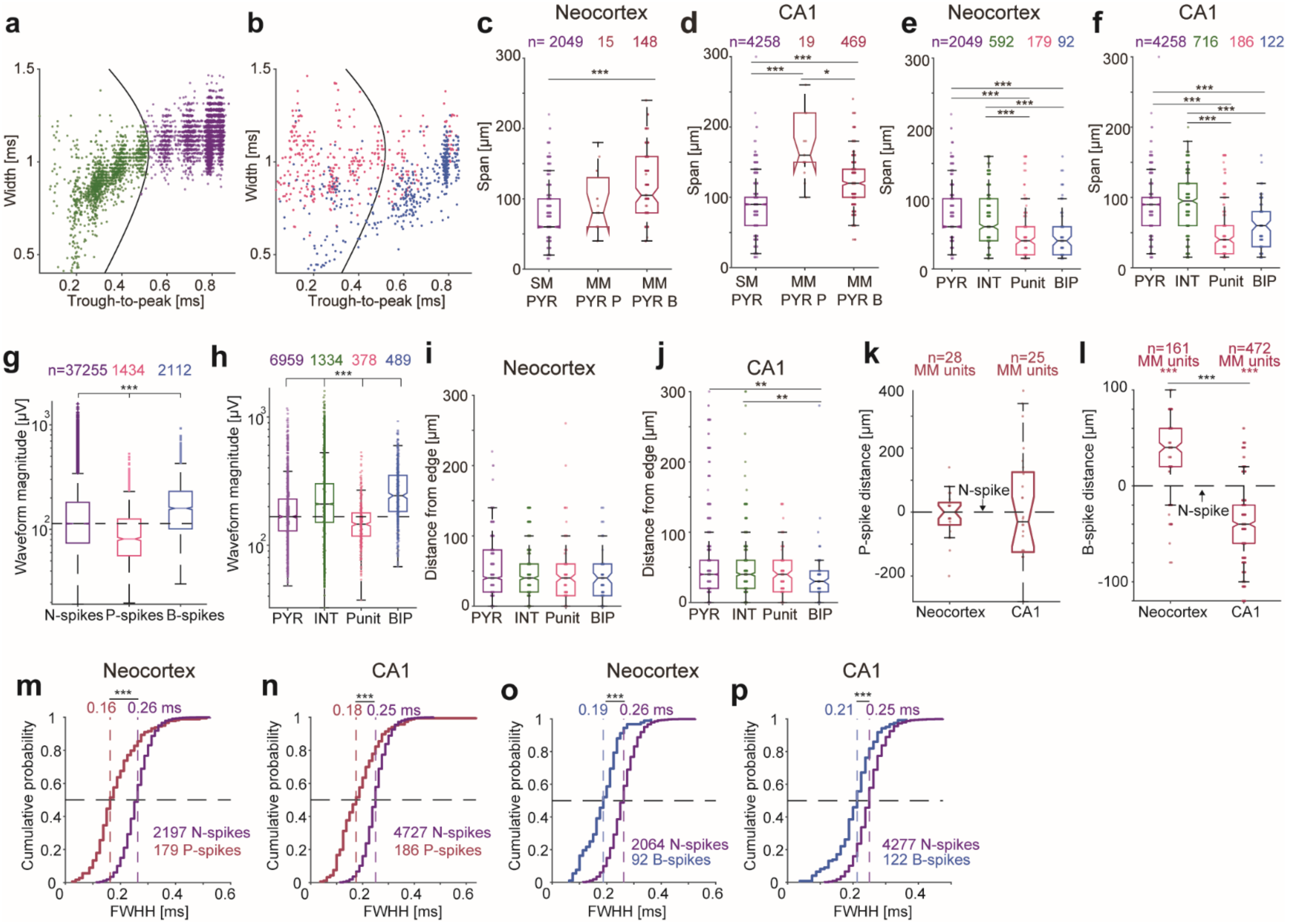

Supplementary Figure 1. Compared with PYRs and INTs, Punits and BIPs are more spatially compact. a PYR and PV-like INT classification. Units with an N-spike on the electrode with the largest trough-to-peak magnitude were classified into PYR (purple) and PV-like INT (green) using a Gaussian mixture model[42]. The classifier uses two features derived from the single-channel waveform, spike width and trough-to-peak duration. **b** Punits (pink) and BIP (blue) plotted in the same feature space as in (**a**). **c-d** SM PYRs are more compact than MM PYRs in neocortex and CA1. Here and in (**e-j**), */**/***: p<0.05/p<0.01/p<0.001, Kruskal-Wallis test, corrected for multiple comparisons. Box plot conventions are the same as in **Fig. 2f**. **e-f** Among SM units, Punits and BIPs are more compact in space than PYRs and INTs in both neocortex and CA1. **g** B-spikes have larger waveform magnitude compared with N-spikes, and N-spikes have larger waveform magnitude compared with P-spikes. **h** Waveform magnitude at main channel for every group. Magnitudes differ between all groups. Specifically, BIP waveform magnitude is larger when compared with PYRs and INTs, whereas Punit waveform magnitude is smaller compared with PYRs and INTs. **i-j** Distance between main channel and closest edge of the recording shank for SM units. Compared to PYRs and INTs, the main channel of SM BIPs in CA1 is more frequently positioned closer to the shank edge in CA1. **k** In neocortex, P-spikes appear above the same-unit N-spike (median [IQR] distance: 20 [-30 30] μm). In CA1, the P-spikes appear below the N-spikes, with a distance of -20 [-140 120] μm. **l** B-spikes appear above the same-unit N-spike in neocortex (40 [20 60] μm). In CA1, B-spikes appear below the same-unit N-spike (-40 [-60 -20] μm). ***: p<0.001, Wilcoxon's test. Lined ***: p<0.001, U-test. **m-p** P- and B-spikes are narrower than N-spikes when using the full-width at half-height (FWHH) metric. ***: p<0.001, U-test.

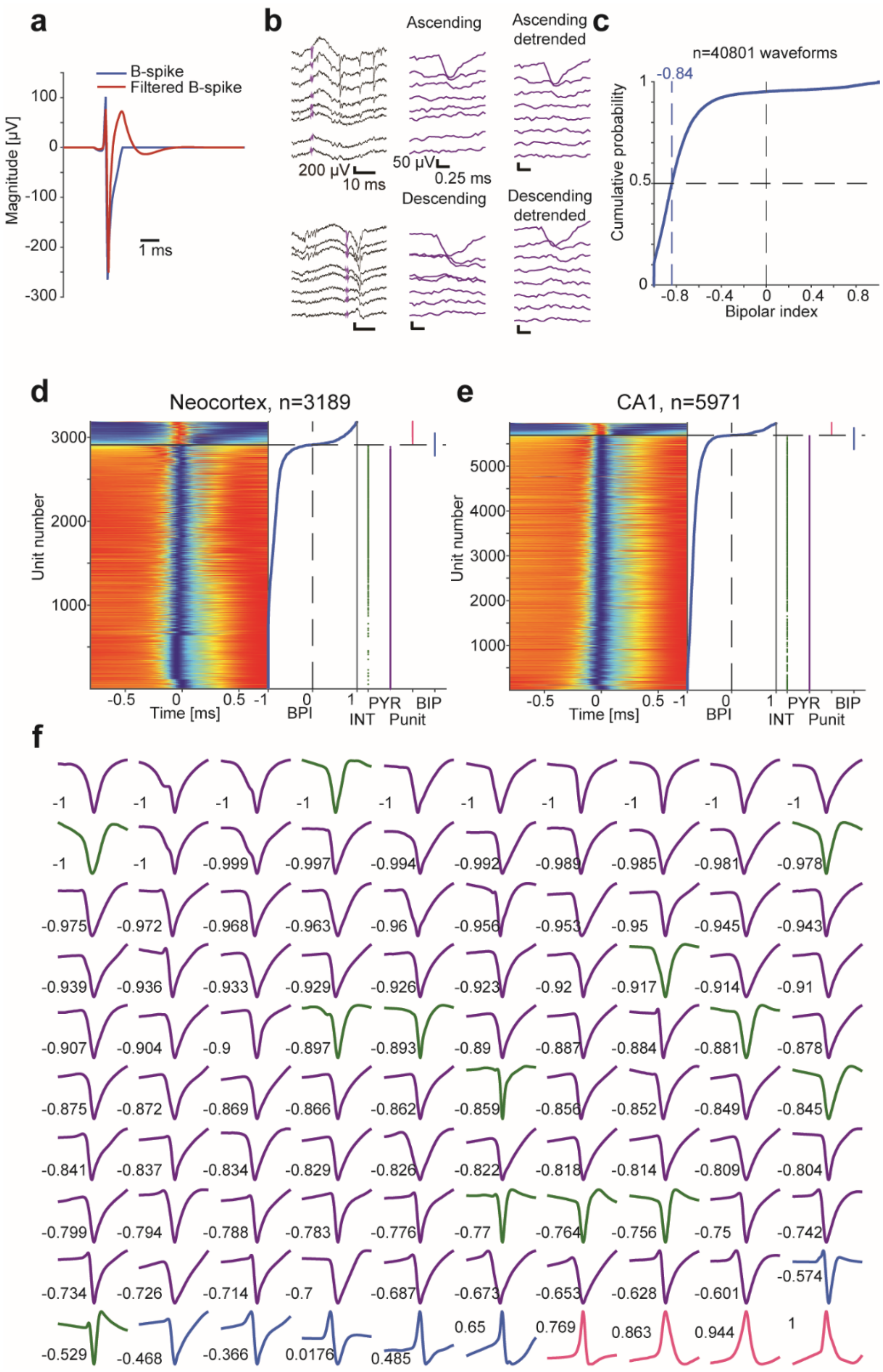

Supplementary Figure 2. Spike waveform categorization. a High-pass filtering transforms a wideband B-spike into a triphasic spike. The specific filter used is a third-order Butterworth, 300-6000 Hz. Triphasic spikes are generated with a wide range of filter settings (e.g., high-pass at 300, 500, 800 Hz; 1st, 2nd, 3rd order). **b** Example of two spikes of the same unit. First row, a spike that occurred during the ascending phase of a gamma cycle. Second row, a spike that occurred during the descending phase of a gamma cycle. In both rows, the left column shows wideband traces; the central column shows the raw spikes; and the right column shows the detrended spikes. **c** Bipolar index (BPI) of the main channel waveforms of all 9160 units in the dataset. Blue dashed line indicates the median BPI. Most waveforms are N-spikes. **d-e** Main channel waveforms. **d** Left, Waveforms of all neocortical units. Middle, BPI of every unit. Right, Classification of each unit. **e** Same, for CA1 units. **f** Waveform examples. A statistically representative set of 100 waveforms was randomly selected from the 9160 units. Numbers indicate BPI, and colors indicate the class of the unit: PYR, purple; INT, green; BIP, blue; Punit, pink.

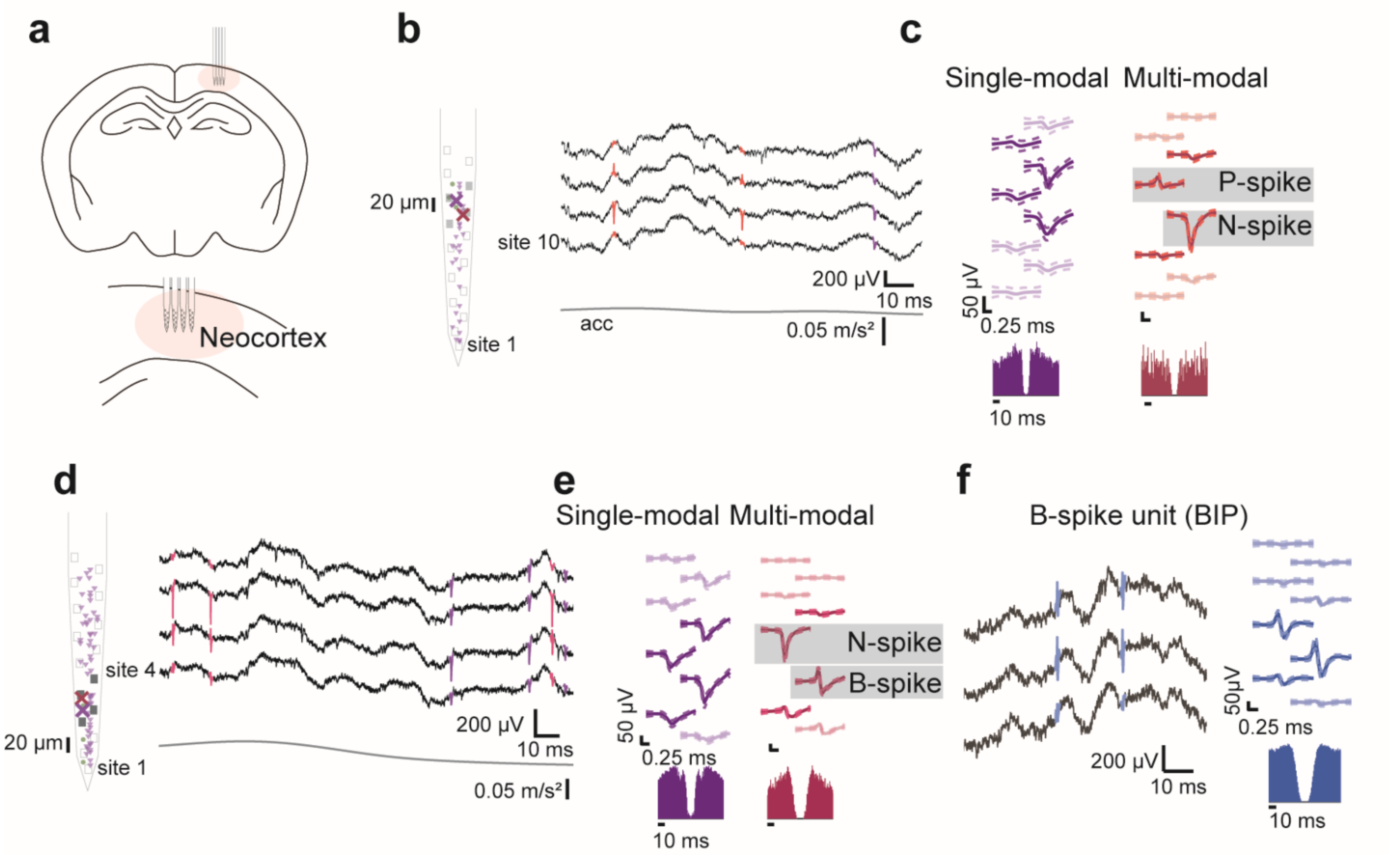

Supplementary Figure 3. Units with P-spikes and B-spikes appear also in neocortex. a Multi-shank recording array in the neocortex. **b** SM PYR and a MM unit with P-spikes recorded from the neocortex. Left, Schematic shank with 32 simultaneously-recorded units. Right, Wideband traces recorded by four adjacent electrodes. All conventions here and in **c** are the same as in **Fig. 1bc. c** Wideband waveforms and ACHs of the units highlighted in (**b**). **d** SM PYR and a MM unit with B-spikes recorded from the neocortex. Left, Schematic shank with 59 simultaneously-recorded units. Right, Wideband traces recorded by four adjacent electrodes. **e** Wideband waveforms and ACHs of the units highlighted in (**d**). **f** B-spikes appear without N-spikes in the neocortex.

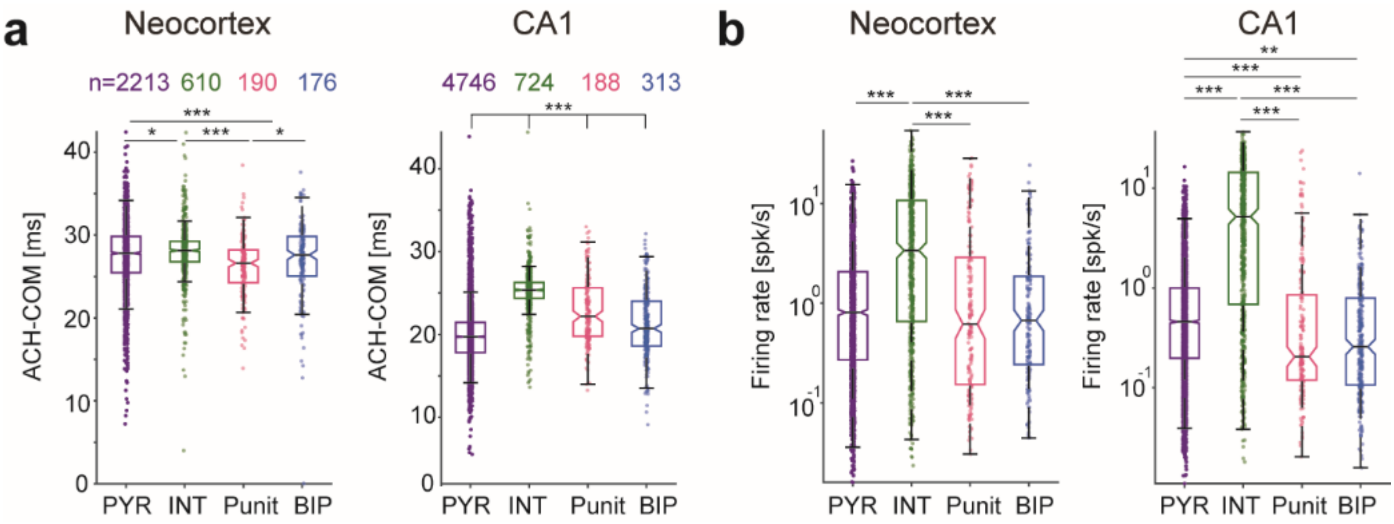

Supplementary Figure 4. CA1 Punits and BIPs are less bursty than PYRs but more bursty than INTs. **a** In the neocortex, Punits are more bursty than INTs and PYRs. In CA1, PYRs are most bursty, followed by BIPs, Punits, and INTs. Sample size is indicated on top and is the same here and in (**b**). Here and in (**b**), ***: p<0.001, Kruskal-Wallis test, corrected for multiple comparisons. Box plot conventions are the same as in **Fig. 2f**. **b** In both neocortex and CA1, the firing rates of Punits and BIPs are lower than INTs.

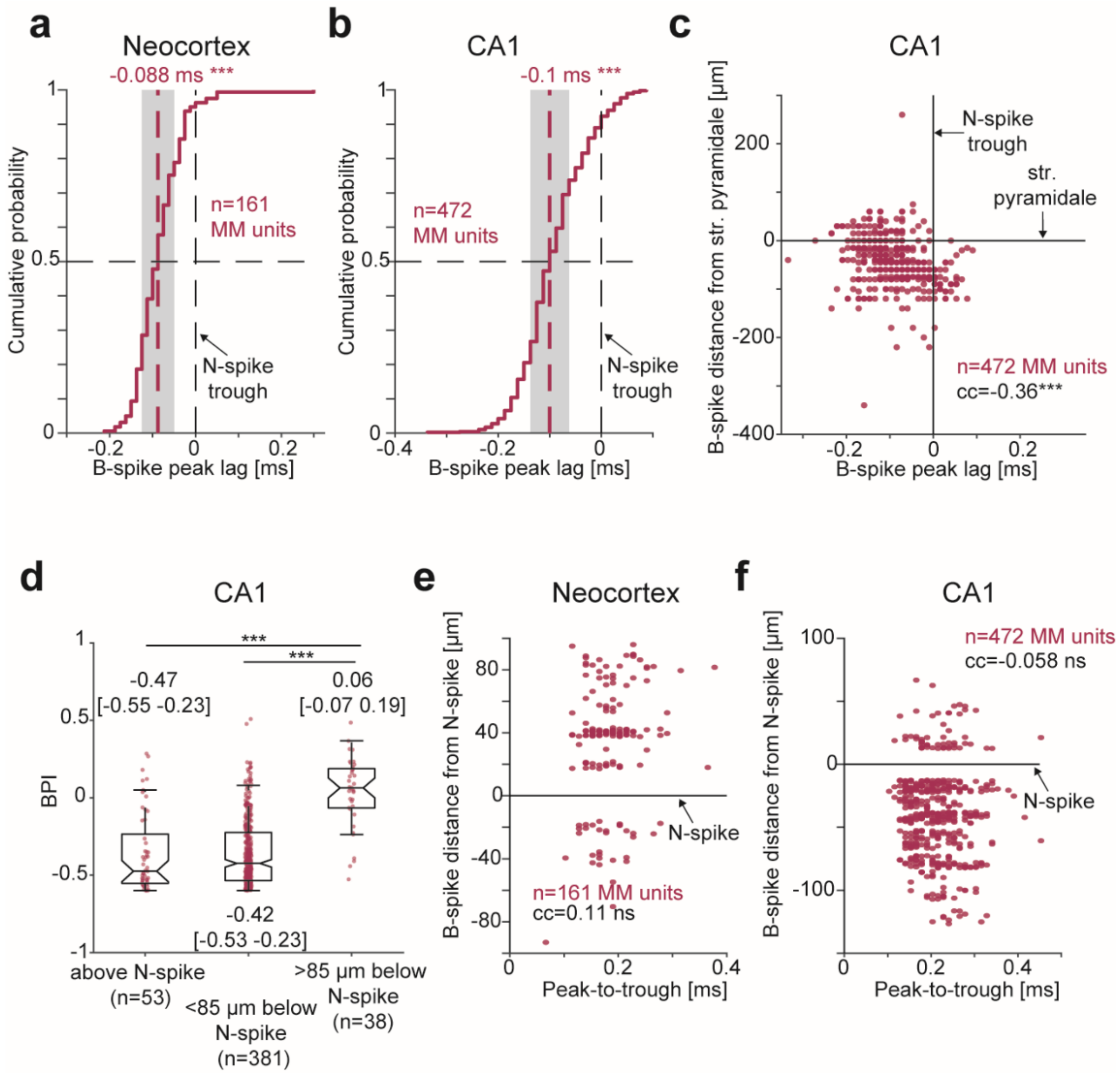

Supplementary Figure 5. B-spike peak precedes same-unit N-spike trough. a-b B-spike peak precedes same-unit N-spike trough. **a** Time lag between B- and N-spikes in neocortical MM units. Here and in (**c**): ***, p<0.001, Wilcoxon's test comparing to a zero null. Grey patch, 95% confidence limits. **b** Time lag between B- and N-spike in CA1 MM units. **c** Distance of B-spike from the center pf CA1 str. pyramidale vs. B-spike peak lag from N-spike trough. Distances are positive when the B-spike is closer to the surface of the brain. cc, rank correlation coefficient; ***: p<0.001, permutation test. **d** Biphasic index (BPI) vs. distance between B- and N-spikes. ***: p<0.001, Kruskal-Wallis test. Box plot conventions are the same as in **Fig. 2f**. **e-f** Distance of B-spike from and N-spike vs. B-spike peak-to-trough duration in neocortex (**e**) and in CA1 (**f**).

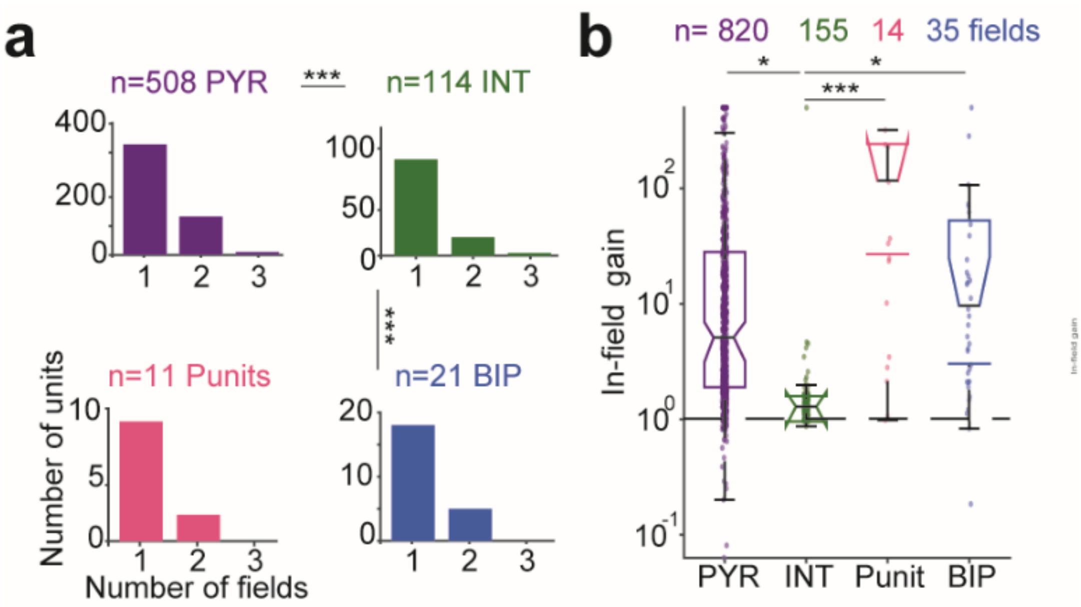

Supplementary Figure 6. BIP gain and number of fields are not consistently different from PYR. a Number of place fields per unit. ***: p<0.001, G-test, corrected for multiple comparisons. Error bars, SEM. **b** In-field gain. */***: p<0.05/p<0.001, Kruskal-Wallis test. Box plot conventions are the same as in **Fig. 2f**.
